# Causal relationships between circulating inflammatory factors and IgA vasculitis: a bidirectional Mendelian randomization study

**DOI:** 10.3389/fimmu.2023.1248325

**Published:** 2023-09-11

**Authors:** Jiading Qin, Ling Zhang, Bo Ke, Tingting Liu, Chunfang Kong, Chenghao Jin

**Affiliations:** ^1^ Medical College of Nanchang University, Nanchang, China; ^2^ Department of Hematology, Jiangxi Provincial People’s Hospital, The First Affiliated Hospital of Nanchang Medical College, Nanchang, China; ^3^ Key Biologic Laboratory of Blood Tumor Cell of Jiangxi Province, Jiangxi Provincial People’s Hospital, Nanchang, China; ^4^ National Clinical Research Center for Hematologic Diseases, the First Affiliated Hospital of Soochow University, Soochow, China

**Keywords:** bidirectional, C-reactive protein, circulating inflammatory regulators, Mendelian randomization, IgA vasculitis

## Abstract

**Background:**

IgA vasculitis (IgAV) is an immune-associated vasculitis, yet its exact etiology remains unclear. Here, we explore the interaction between IgAV and inflammatory factors using bidirectional Mendelian randomization (MR).

**Methods:**

We conducted a bidirectional summary-level MR analysis to delineate the causality of C-reactive protein (CRP), procalcitonin (PCT), and 41 circulating inflammatory regulators with IgAV. Data on genetic variants related to inflammation were obtained from three genome-wide association studies (GWASs) on CRP, PCT, and human cytokines, whereas data on IgAV was from large meta-analyses of GWAS among 216 569 FinnGen Biobank participants. The primary MR analysis was performed using the inverse-variance weighted (IVW) approach, and the sensitivity analyses were carried out using MR-Egger, weighted median, weighted mode, and MR-pleiotropy residual sum and outlier.

**Results:**

This study revealed the association of CRP higher levels with increased risk of IgAV through IVW method (Estimate odds ratio [OR] = 1.41, 95% confidence interval [CI]: 1.01-1.98, *P* = 0.04), MR-Egger (OR = 1.87, CI: 1.15-3.02, *P* = 0.01), weighted median (OR = 2.00, CI: 1.21-3.30, *P* = 0.01) and weighted mode (OR = 1.74, CI: 1.13-2.68, *P* = 0.02). Furthermore, elevated IL-8 was strongly implicated with a higher risk of IgAV (IVW OR = 1.42, CI: 1.05-1.92; *P* = 0.02). Conversely, genetically predicted IgAV was associated with decreased levels of TNF-β (IVW estimate *β* = -0.093, CI: -0.178 - -0.007; *P* = 0.033). Additionally, no such significant statistical differences for other inflammatory factors were found.

**Conclusion:**

Our current study using bidirectional MR analysis provides compelling evidence for a causal effect of CRP, PCT, and circulating inflammatory regulators on IgAV. These findings contribute to a better understanding of the pathogenesis of IgAV and emphasize the potential of targeting inflammatory factors for therapeutic interventions.

## 1 Introduction

IgA vasculitis (IgAV), also known as Henoch-Schönlein purpura, is an immune-associated vasculitis characterized by IgA dominant immune complex deposited within or around the small vessels, manifested as palpable purpura, abdominal pain, arthritis, and renal involvement (1). IgAV is the most common vasculitis in children, yet it can also affect adults, with more severe systemic symptoms and the potential for recurrent episodes (2). The pathogenesis of IgAV is far from clear. Evidence has revealed that several environmental or infectious factors are associated with it. For example, streptococcal infection has been considered the most common infectious trigger for childhood IgAV, and it is related to the clinical phenotypes and relapse/recurrence of IgAV (3). Another factor is heat shock proteins (HSPs), some of which show an antiviral effect by inhibiting viral proliferation and activating immune pathways to counteract IgAV during viral infection (4). However, environmental and infectious factors are accompanied by changes in cytokine profiles (5), and the latter’s role in the etiology of IgAV is currently unclear.

Inflammation can disrupt tissue cells, increasing capillaries’ fragility and permeability, thereby resulting in extravasation of blood and the development of purpura (6). The progression of IgAV involves multiple organs, including the kidney, skin, bone, and abdomen, manifesting with a diverse range of symptoms. Corticosteroids, considered anti-inflammatory hormones, can benefit patients with complex symptoms of IgAV in their early treatment (7). However, some patients often miss the optimal treatment window due to late intervention, leading to recurrent episodes in some cases and further progression to end-stage kidney disease in others (8). Previous reports have proposed that serum levels of TNF-α, IL-23, IL-18, IL-17A, IL-8, and IL-6 are upregulated in patients with IgAV than those in control donors, whereas the negative regulators TGF-β, IL-10, and IL-27 were significantly lower (9–11). Some researchers speculated that these circulating inflammatory factors are essential in the pathogenesis of IgAV, emphasizing their predictive value for IgAV.

The possible mechanism of IgAV is currently inconclusive. Some cytokines, especially IL-8, have been reported to be involved in the pathogenesis of IgAV. IL-8 is a chemokine central in recruiting neutrophils, the primary effector cells in IgAV (12). IL-8 can be induced by IgA immune complexes, formed by galactose-deficient IgA1 and anti-IgA1 antibodies (13). Interestingly, these immune complexes activate the complement system, such as C3a and C5a, enhancing the expression of IL-8 and other pro-inflammatory cytokines, such as MCP-1, E-selectin, and ICAM-1 (14). Furthermore, these cytokines stimulate neutrophil recruitment, and the activated neutrophils cause extensive damage to the vascular endothelium via antibody-dependent cellular cytotoxicity, complement-mediated cytotoxicity, and reactive oxygen species (15), ultimately contributing to vascular leakage, edema, hemorrhage, and tissue necrosis, manifested by purpura, arthritis, abdominal pain, and renal involvement.

Despite previous epidemiological, genomic, and biological studies that have established a particular link between IgAV and circulating inflammatory factors (16), there has been a lack of research elucidating the causal relationship in the whole picture. As is widely recognized, inflammation factors represent a dynamic process, and observing their levels at a specific time point in clinical settings may not accurately reflect the overall inflammatory status throughout the disease. Based on an observational study involving 200 patients with IgAV who underwent hemoperfusion or glucocorticoid, distinct cytokine alternations were observed across different clinical responses in IgAV (17). Moreover, the issue highlights the importance of compliance and accuracy in clinical measurement. Similarly, we cannot eliminate the reciprocal impact when assessing the autonomous effect of a single alteration in serum cytokine levels. In addition, the relationship between circulating inflammatory factors and IgAV is ambiguous. A study has suggested that polymorphism of IL-8 is associated with nephritis in children with IgAV (18). In comparison, another study has demonstrated no interaction between the two in childhood, regardless of the joint, gastrointestinal, and renal manifestations (19). An observational study has declared that procalcitonin (PCT) is significantly associated with gastrointestinal complications, whereas C-reactive protein (CRP) is not a specific predictor for different clinical manifestations of IgAV (20). However, another study has revealed that CRP has significant clinical value in IgAV with gastrointestinal involvement (21). Therefore, designing a comprehensive and precise experiment to evaluate the association between circulating inflammatory factors and the risk of IgAV disease and their relative contributions is necessary.

Observational studies, commonly employed to investigate the association between an exposure and an outcome, are susceptible to confounding and bias, posing a risk of yielding erroneous findings. In contrast, Mendelian randomization (MR), a technique utilizing genetic variants as instrumental variables, emerges as an alternative approach to mitigate confounders and bias and furnish robust evidence for causality (22). In contrast to single-sample MR, two-sample MR triumphs in mitigating bias and confounding by leveraging summary statistics gathered from various studies. Furthermore, many studies use it to unravel more precise estimates of causal effects between circulating inflammatory factors and others through augmented sample sizes and enhanced statistical power (23–25). Therefore, embarking on a captivating bidirectional exploration through MR utilizing comprehensive genome-wide association summary-level data, we sought to unravel the intricate associations between IgAV and inflammatory cytokines containing CRP, PCT, and circulating inflammatory regulators.

## 2 Materials and methods

### 2.1 Study protocol and data source

**Figure 1:**
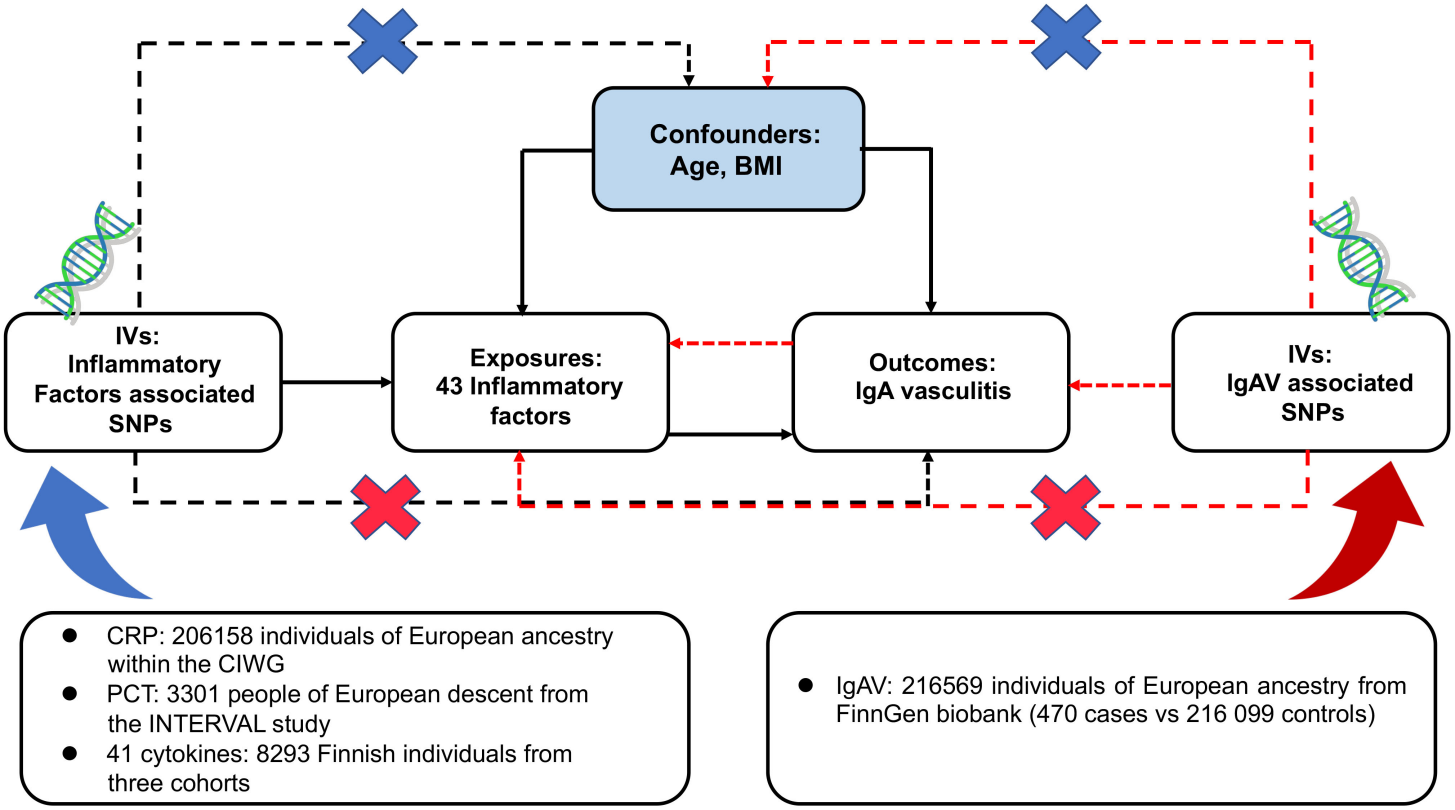
Assumptions and study design of the bidirectional MR study of the associations between 43 circulating inflammatory factors and IgAV. BMI, body mass index; CIWG, Cohorts for Inflammation Work Group; IVs, instrumental variables; SNPs, single-nucleotide polymorphisms.

The design scheme of bidirectional MR is shown in **Figure 1**. MR depends on three major assumptions (1): the genetic variant chosen as the instrumental variable is strongly implicated with the exposure (2); the genetic variant is not related to confounders (3); genetic variants influence the outcome only through the exposure (26). This work utilized summary-level data from known genome-wide associated studies (GWAS) of 41 circulating inflammatory regulators, CRP, PCT, and IgAV. First, we deduced the causal relationship between circulating inflammatory factors and IgAV by selecting genetic variants of circulating inflammatory factors. Subsequently, the causal relationship between IgAV and each circulating inflammatory factor was deduced by choosing genetic variants associated with IgAV.

### 2.2 Genetic instrumental variables for circulating inflammatory factors

Genetic variants of CRP and PCT were collected from a meta-analysis of GWAS containing 206 158 unities of European ancestry within the Cohorts for Inflammation Working Group (CIWG) (27) and 3 301 persons of European descent from the INTERVAL study (28), respectively. The cohort of CRP contains 2.4 million genetic variants compared with 10.5 million in PCT. Furthermore, to validate the causal relationship between CRP and IgAV, we obtained another cohort with 20 623 European individuals containing 8.0 million SNPs (29). Genetic associations above genetic variants and log-transformed CRP were adjusted for demographic structure.

Genetic predictors for 41 circulating inflammatory regulators were derived from the most comprehensive cytokine-related GWAS meta-analysis of three independent cohorts, containing 8 293 Finnish participants from the Cardiovascular Risk in Young Finns Study (YFS) and the “FINRISK” studies (FINRISK1997 and FINRISK2002) (30). A two-step inverse transformation was conducted to normalize the distribution of these 41 regulators. An additional genetic model with the adjustment for age, gender, body mass index (BMI), and the first ten genetic principal components was conducted to evaluate univariable associations between 41 regulator concentrations and 10.7 million genetic polymorphisms.

### 2.3 Genetic instrumental variables for IgAV

Summary-level data on IgAV was obtained from a GWAS containing 470 cases and 216 099 controls of European ancestry from the FinnGen biobank. FinnGen project, initiated in Finland in 2017, is an independent study that combines genomic information with digital health care data (31), and IgAV was defined by ICD-9 and ICD-10 codes. The BOLT-linear mixed model (BOLT-LMM) was suitable for GWAS in large cohorts (32). The GWAS was conducted in BOLT-LMM and regulated for gender and genotype array. Since the BOLT-LMM association statistics were linear, we converted them to a logarithmic odds ratio through a reasonable approximation log OR ≈ *β*/(*μ*[1-*μ*]) for the genetic correlations with IgAV, where *β* is the reported effect size from the BOLT-LMM and *μ* represents the case fraction for the binary trait. The corresponding SE was divided by *μ*(1-*μ*) (33). After that, we applied two-sample MR methods using GWAS summary statistics to deduce the causality between IgAV and circulating inflammatory factors. No overlap was observed because CRP, PCT, circulating inflammatory regulators, and IgAV specimens were acquired from distinction consortiums.

### 2.4 Genetic instrumental variables selection

To conform to the MR conceptions (**Figure 1**), all SNPs are independently and strongly (*R* ^2^ < 0.001) predicted exposures from the FinnGen biobank or GWAS at genome-wide significant (GS) (*P* < 5 x 10^-8^). Due to only ten circulating inflammatory factors and CRP having ≥ 3 unique SNPs that reached GS and no GS SNPs for PCT and IgAV, a less stringent threshold of 1 x 10^-5^ was adopted to collect more SNPs for PCT, circulating inflammatory factors, and IgAV.

Furthermore, using the available GWAS summary data, we evaluated whether any SNPs were related to confounders (age at recruitment and BMI) and outcomes at a P-value of Bonferroni level (0.05/number of SNPs). BMI was reported as an independent risk factor in patients with IgAV (34), and it could accelerate the course of IgAV (8, 35). Moreover, obesity could lead to chronic low-grade inflammation (36). Thus, the BMI should be regarded as a confounder. We obtained the correlations of those SNPs with BMI from a meta-analysis of GWAS in around 700 000 individuals of European ancestry (37). IgAV is often found in childhood, whereas adults have more severe symptoms and worse outcomes (2), contributing to survival bias (38). To mitigate the bias, we excluded SNPs associated with survival proxied by age at recruitment, as previously described (24). Finally, we assessed the strength of each SNP using the *F*-statistic, a function of the magnitude and precision of the genetic effect on the trait: *F* = *R* ^2^(N - 2)/(1-*R* ^2^), where *R* ^2^ represents the proportion of the trait variance explained via the SNP and N represents the sample size of the GWAS of SNPs with the trait (39). The formula *R* ^2^ = 2 x EAF x (1-EAF) x *β* ^2^ was used to determine the values of *R* ^2^, where EAF represents the effect allele frequency (EAF) of the SNP, and *β* represents the determined effect of SNP on trait (40). SNPs with *F* < 10 were excluded because they suggested insufficient strength to guarantee the availability of the SNPs.

### 2.5 Bidirectional Mendelian randomization analysis

We carried out a bidirectional two-sample MR approach using summary relation data to investigate the causal direction of relationships between IgAV and inflammation regulators. Details of SNPs, effect sizes, alleles, SEs, EAF, and *P* values are required for our analyses (41). Unavailable SNPs in the datasets were displaced by proxies at *R* ^2^ > 0.9 in Ldlink (42).

We rigorously enforced data harmonization to ensure that the effect of SNPs on the outcome and exposure corresponded to the same allele. For SNPs with varying effect alleles induced by different strands, strand correction was performed to guarantee the same effect allele. In addition, we excluded palindromic SNPs, which are more challenging to harmonize due to the identical alleles on both strands, to avoid ambiguity based on whether exposure and outcome GWAS reported the same effect allele. We summarized the estimates in the significant analysis using the inverse-variance weighted (IVW) with multiplicate random effects approach, which supplies a concise estimation and considers potential heterogeneity among the Wald ratio calculated from SNPs (43). The influences in 41 regulators were reported as changes in inverse normalized cytokines concentration per effect allele dosage. CRP and PCT were reported as changes in their natural log-transformed concentration per effect allele dosage. Findings of the 41 cytokines effect and CRP or PCT impact on IgAV were showed as ORs (95% CIs) per 1 SD genetic predicted cytokine change and per 1% genetic predicted CRP or PCT change, respectively. The influences of IgAV on circulating inflammatory factors were reported as *β* coefficient and 95% CIs.

### 2.6 Sensitivity analysis

A sensitivity analysis set containing MR-Egger, weighted median, weighted mode, and Mendelian Randomization Pleiotropy Residual Sum and Outlier (MR-PRESSO) was performed. MR-Egger analysis supplies an estimation of instrumental variable pleiotropy with a nonzero intercept, indicating the bias in IVW assessment (44). The weighted median method that chooses the median MR considered as the causal estimate was taken into account for multiple genetic variants to be invalid or present pleiotropy (45). MR-PRESSO utilizes a global test to evaluate horizontal pleiotropy and could correct for potential pleiotropic outliers (46). The Cochran Q test and *I* ^2^ index were used to determine heterogeneity in the IVW estimates.

The Bonferroni approach was utilized to correct the multiple testing, and a *P*-value below 0.05 was considered a significant association. Data analyses were performed using MRPRESSO (version 1.0) or TwoSampleMR (version 0.5.6) packages in R software (version 4.3.0), all of which were two-sided. Reporting follows the STROBE-MR statement.

## 3 Results

### 3.1 Bidirectional causal effect between IgAV and CRP

**Figure 2:**
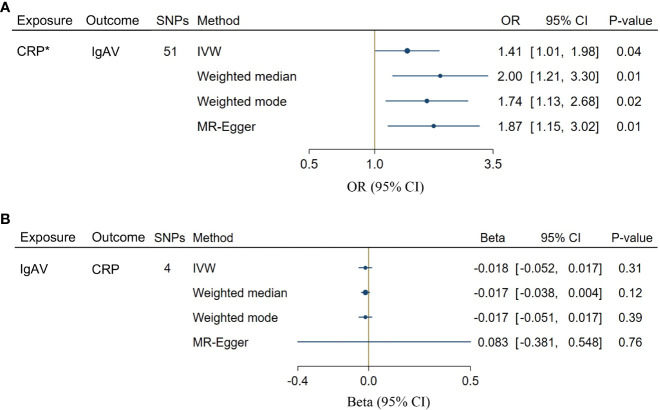
Bidirectional causal associations between CRP and IgAV. **(A)** The causal effect of CRP on IgAV. **(B)** The causal effect of IgAV on CRP. Asterisk (*) indicates the SNPs were associated with exposure at a genome-wide significant level. Exposures without asterisk indicate the SNPs were associated with exposure at P < 1 x 10^-5^ level. IVW, inverse variance weighting.

We chose 51 SNPs genome-wide related to CRP (**Supplementary Table S1**). Genetically predicted CRP levels were implicated with the risk of IgAV using GS SNPs under inverse-variance weighted (IVW) [OR = 1.41, CI: 1.01-1.98; *P* = 0.04], weighted median [OR = 2.00, CI: 1.21-3.30; *P* = 0.01], weighted mode [OR = 1.74, CI: 1.13-2.68; *P* = 0.02] as well as MR-Egger [OR = 1.87, CI: 1.15-3.02; *P* = 0.01] (**Figure 2A**), indicating solid shreds of evidence for the causal effect of CRP on IgAV. Whereas there was no evidence for the causal impact of IgAV on CRP with the less stringent cut-off (*P* = 0.31) using 4 SNPs (*P* < 1 x 10^-5^, **Supplementary Table S2**), and the result of other MR methods also indicated no statistical significance (**Figure 2B**). No horizontal pleiotropy and heterogeneity were observed based on the causal influence of CRP on IgAV (*P*_Q_ = 0.45, *P*_MR-PRESSO_ = 0.47), suggesting the former result was robust and reliable (**Supplementary Table S3**). However, a significant horizontal pleiotropy was observed on the causal effect of IgAV on CRP (*P*_MR-PRESSO_ = 0.008), and heterogeneity was also detected with *P*_Q_ < 0.001, indicating the latter result was unreliable.

**Figure 3:**
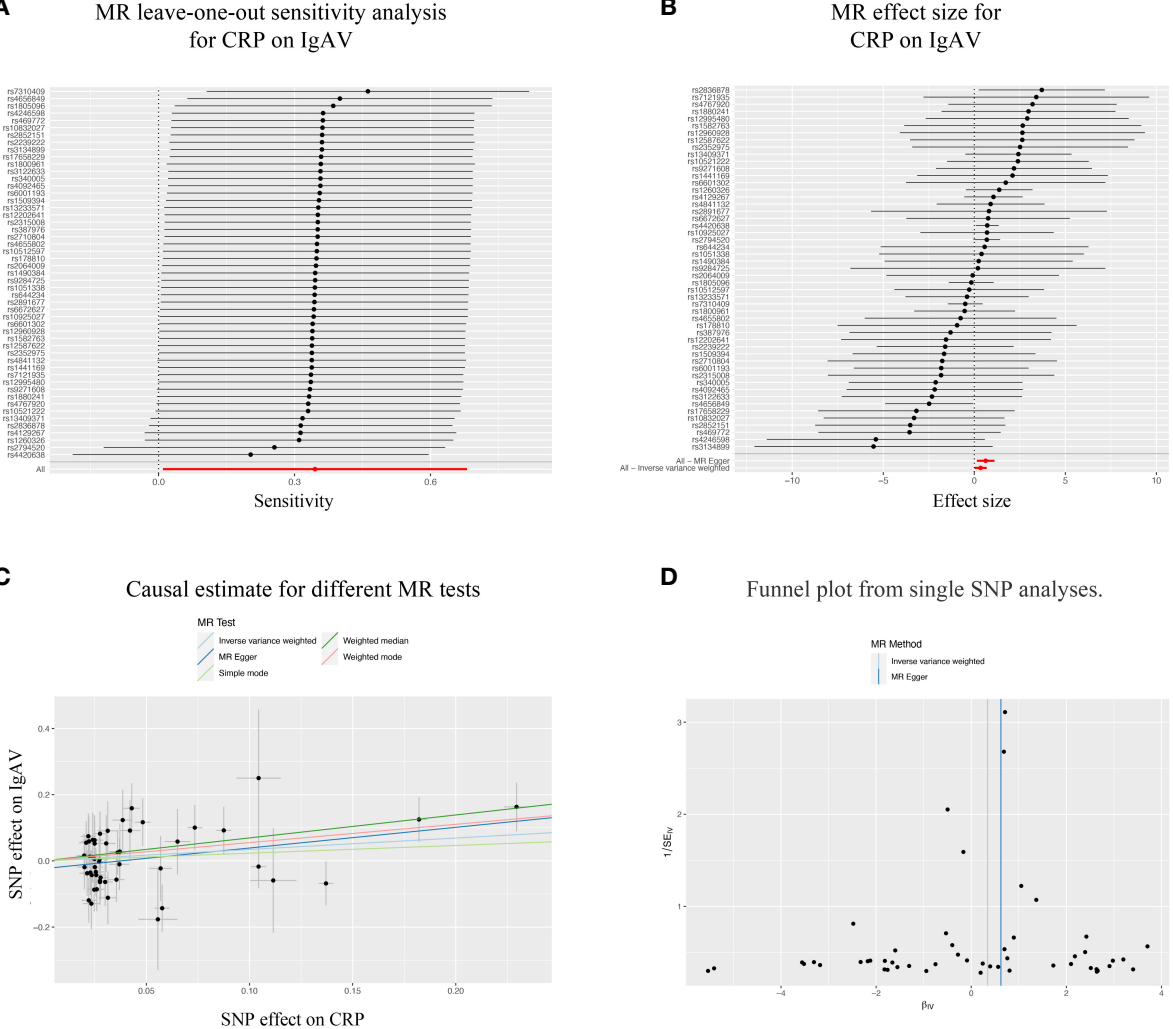
Sensitivity analysis of the bidirectional causal associations between CRP and IgAV. **(A)** MR leave-one out sensitivity analysis for CRP on IgAV. **(B)** MR effect size for CRP on IgAV. **(C)** Causal estimate for different MR tests. **(D)** Funnel plot from single SNP analyses.

Considering the significant causal effects of CRP on IgAV, we conducted a leave-one-out sensitivity analysis for each SNP on the causal effect of CRP on IgAV. We found the result was always at one side of the zero line, no matter whether any SNP was excluded (**Figure 3A**). Moreover, the effect size of IVW and MR-Egger was visualized with 51 SNPs, and we observed that these two results were consistent with significance (**Figure 3B**). Additionally, the trends of different MR tests were relatively uniform with significance (**Figure 3C**). At last, funnel plot analyses showed that the distribution of individual SNPs is evenly spread on both sides of the vertical line in either IVW or MR-Egger analysis (**Figure 3D**).

Furthermore, we utilized a cohort with a smaller sample size to verify the significant causal effects of CRP on IgAV, and acquired 5 SNPs genome-wide related to CRP (**Supplementary Table S4**). Genetically predicted CRP levels were implicated with the risk of IgAV using GS SNPs under the IVW method [OR = 2.10, CI: 1.22-3.60; *P* = 0.01] (**Supplementary Figure 1A**). We also found that regardless of excluding any SNP, the result was always on one side of the zero line (**Supplementary Figure 1B**). Moreover, the effect of IVW and MR-Egger was visualized using these 5 SNPs, demonstrating that IVW was on one side of the zero line, and the effect of MR-Egger was roughly the same (**Supplementary Figure 1C**). Similarly, the trends of different MR tests were also relatively uniform with significance (**Supplementary Figure 1D**). No horizontal pleiotropy and heterogeneity were observed based on the causal influence of CRP on IgAV (*P*_Q_ = 0.74, *P*_MR-PRESSO_ = 0.84), indicating the reliable result in the causal effects of CRP on IgAV based on the validation cohort.

### 3.2 Bidirectional causal effect between IgAV and PCT

**Figure 4:**
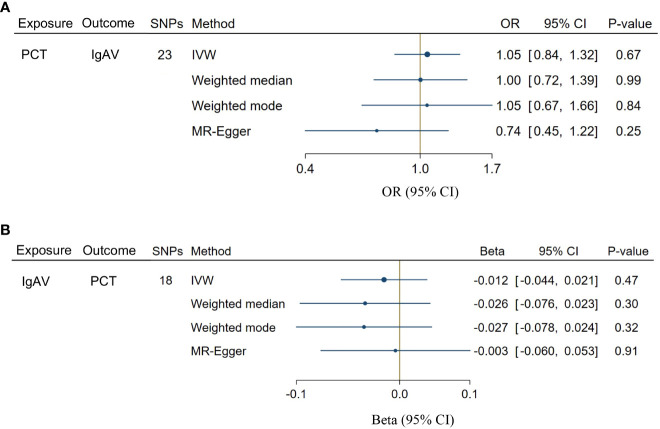
Bidirectional causal associations between PCT and IgAV. **(A)** the causal effect of PCT on IgAV. **(B)** the causal effect of IgAV on PCT. IVW, inverse variance weighting.

A total of 23 SNPs implicated with PCT were selected at a less stringent cut-off (*P* < 1 x 10^-5^, **Supplementary Table S5**), demonstrating the no association between genetically predicted PCT levels and the risk of IgAV (*P* = 0.67, **Figure 4A**). In addition, no evidence showed the causal influence of IgAV on PCT (*P* = 0.47, **Figure 4B**) using 18 SNPs (**Supplementary Table S6**). The findings of other MR approaches were similar to the IVW method (**Figure 4**). MR-PRESSO determined no horizontal pleiotropy, and no heterogeneity was observed in estimating the effect of PCT on IgAV or the impact of IgAV on PCT (**Supplementary Table S7**).

### 3.3 Causal effect of circulating inflammatory regulators on the risk of IgAV

**Figure 5:**
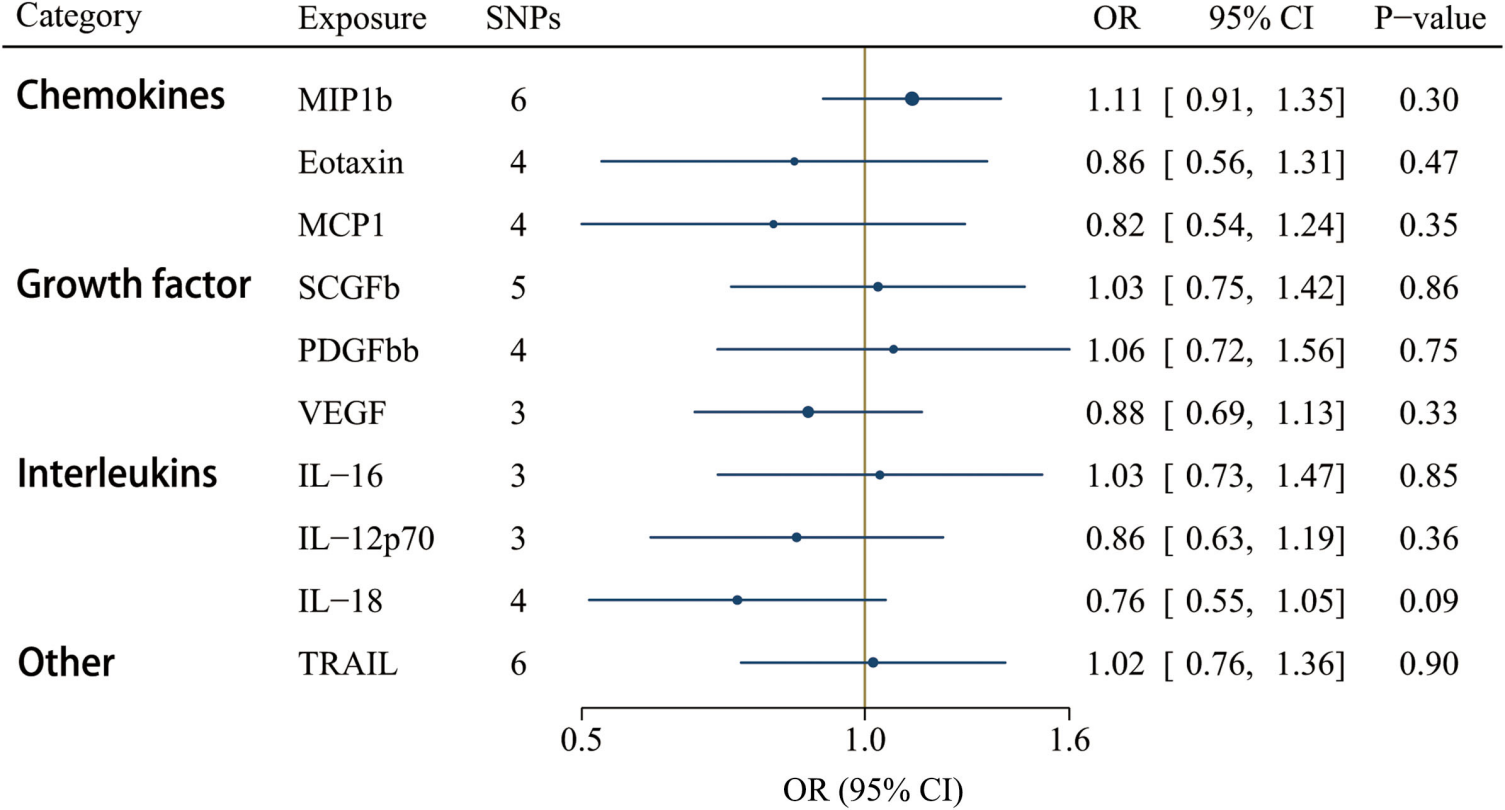
Associations between genetically predicted circulating inflammatory regulators and IgAV (with genome-wide significant SNPs). IL, interleukin; MCP1, monocyte chemotactic protein-1; MIP1b, macrophage inflammatory protein-1β; PDGFbb, platelet-derived growth factor BB; SCGFb, stem cell growth factor beta; TRAIL, TNF-related apoptosis-inducing ligand.

GS SNPs (*P* < 5 x 10^-8^) for ten circulating inflammatory factors represent robust instruments, and the *F*-statistics range from 17.86 to 29513.58 (**Supplementary Table S8**). **Figure 5** displayed the influences of ten circulating inflammatory factors predicted by GS SNP on the risk of IgAV, indicating none of them were related to IgAV in any analyses of multiple comparisons. Additionally, no horizontal pleiotropy and heterogeneity were detected (**Supplementary Table S10**).

**Figure 6:**
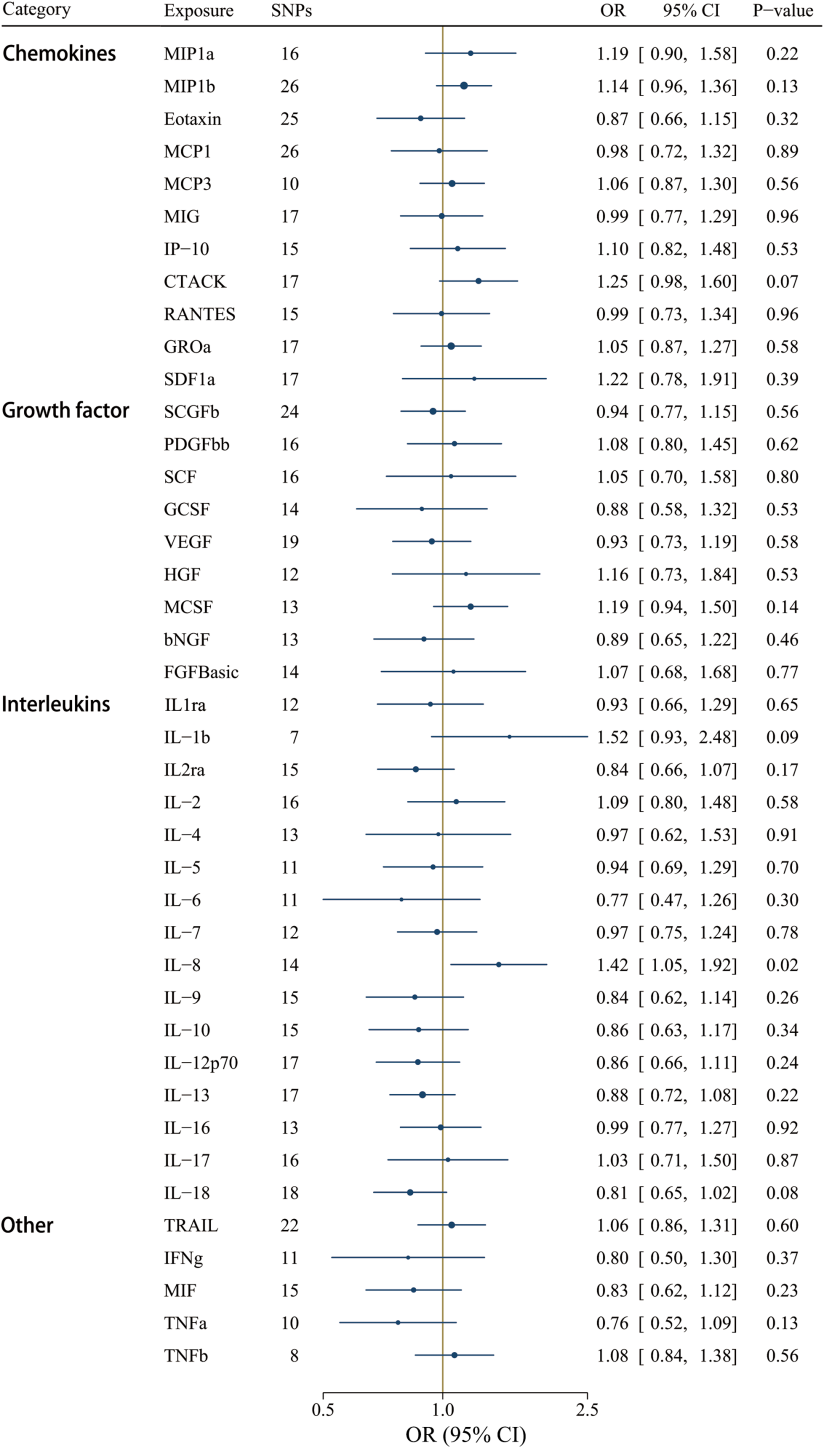
Associations between genetically predicted circulating inflammatory regulators and IgAV (with SNPs reaching P < 1 x 10^-5^). bNGF, beta nerve growth factor; CTACK, cutaneous T-cell attracting chemokine; FGFBasic, basic fibroblast growth factor; GCSF, granulocyte colony-stimulating factor; GROa, growth-regulated oncogene-a; HGF, hepatocyte growth factor; IFNg, interferon gamma; IL, interleukin; IP-10, interferon-gamma-induced protein 10; MCP1, monocyte chemotactic protein-1; MCP3, monocyte-specific chemokine 3; MCSF, macrophage colony- stimulating factor; MIF, macrophage-migration inhibitory factor; MIG, monokine induced by interferon gamma; MIP1a, macrophage inflammatory protein-1a; MIP1b, macrophage inflammatory protein-1b; PDGFbb, platelet-derived growth factor BB; RANTES, regulated on Activation, Normal T Cell Expressed and Secreted; SCF, stem cell factor; SCGFb, stem cell growth factor beta; SDF1a, stromal cell-derived factor-1 alpha; SNPs, single-nucleotide polymorphisms; TNFa, tumor necrosis factor alpha; TNFb, tumor necrosis factor beta; TRAIL, TNF-related apoptosis-inducing ligand; VEGF, vascular endothelial growth factor.

A less stringent cut-off (*P* < 1 x 10^-5^) involved all 41 cytokines, and the *F*-statistics ranged from 12.23 to 29513.58 (**Supplementary Table S9**). Interleukin-8 (IL-8) was the only significant circulating inflammatory regulator associated with IgAV after correcting for multiple comparisons using the IVW method [OR = 1.42, CI: 1.05-1.92; *P* = 0.02] (**Figure 6**). IL-8 could be a detrimental factor in the pathogenesis of IgAV, yet no significant effects were detected using the weighted median, weighted mode, and the MR-Egger methods (**Supplementary Table S11**). However, the magnitude of the IL-8 effect on IgAV was positive and consistent among different MR tests, including the MR-Egger test result [OR = 1.62, CI: 0.87-3.00; *P* = 0.15] (**Supplementary Table S11**). Among these 41 circulating inflammatory regulators, no significant horizontal pleiotropy was revealed by MR-Egger intercept, and no outlier was identified by MR-PRESSO (**Supplementary Table S11**).

### 3.4 Causal effect of IgAV on the levels of circulating inflammatory regulator

**Figure 7:**
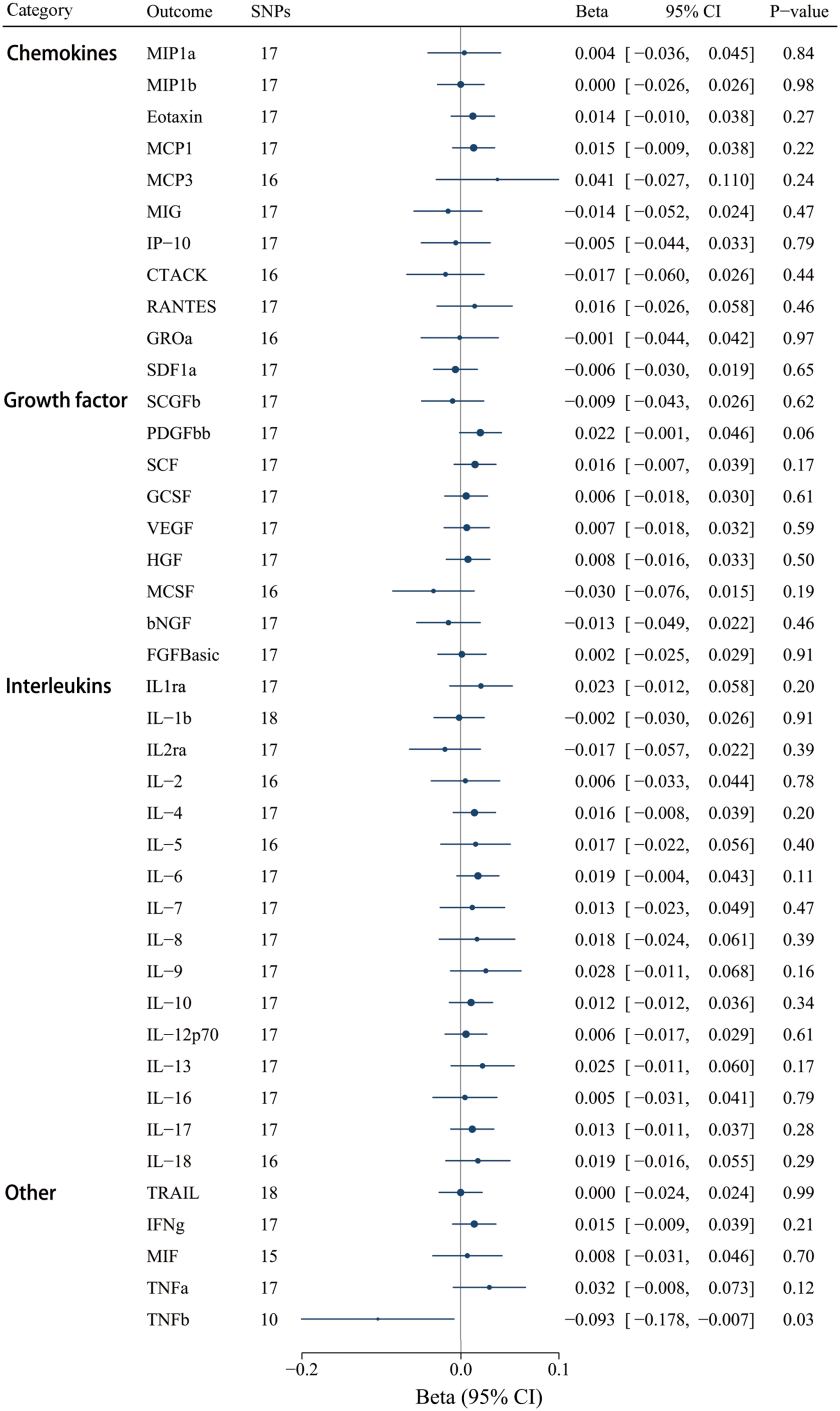
Associations between genetically predicted IgAV and circulating inflammatory regulators. bNGF, beta nerve growth factor; CTACK, cutaneous T-cell attracting chemokine; FGFBasic, basic fibroblast growth factor; GCSF, granulocyte colony-stimulating factor; GROa, growth-regulated oncogene-a; HGF, hepatocyte growth factor; IFNg, interferon gamma; IL, interleukin; IP-10, interferon-gamma-induced protein 10; MCP1, monocyte chemotactic protein-1; MCP3, monocyte-specific chemokine 3; MCSF, macrophage colony- stimulating factor; MIF, macrophage-migration inhibitory factor; MIG, monokine induced by interferon gamma; MIP1a, macrophage inflammatory protein-1a; MIP1b, macrophage inflammatory protein-1b; PDGFbb, platelet-derived growth factor BB; RANTES, regulated on Activation, Normal T Cell Expressed and Secreted; SCF, stem cell factor; SCGFb, stem cell growth factor beta; SDF1a, stromal cell-derived factor-1 alpha; SNPs, single-nucleotide polymorphisms; TNFa, tumor necrosis factor alpha; TNFb, tumor necrosis factor beta; TRAIL, TNF-related apoptosis-inducing ligand; VEGF, vascular endothelial growth factor.

Moreover, we selected 18 SNPs robustly and independently related to IgAV (*P* < 1 x 10^-5^) to assess reverse causation effects. Certain cytokine SNPs remained inaccessible despite using surrogate SNPs and the harmonization process. Thus, we chose different numbers of SNPs for diverse cytokines (**Supplementary Table S12**). The findings revealed that a genetically higher risk of IgAV was associated with decreased TNF-β using the IVW method [*β*_IVW_ = -0.093, CI: -0.178 - -0.007; *P* = 0.033], whereas no other significant correlations were determined (**Figure 7**). Similar to the above result of IL-8, null causal effects of IgAV risk on TNF-β level were found in weighted median (*P* = 0.233), weighted mode (*P* = 0.645), or the MR-Egger method (*P* = 0.262) (**Supplementary Table S13**). Among these 41 circulating inflammatory regulators, neither MR-PRESSO nor MR-Egger intercept demonstrated any significance after correcting for multiple comparisons (**Supplementary Table S13**).

## 4 Discussion

Our present study utilized genome-wide association summary-level data to perform a comprehensive bidirectional MR analysis, thereby evaluating the causal link between CRP, PCT, 41 circulating inflammatory regulators, and IgAV. By employing the bidirectional analytical approach, we could differentiate between upstream and downstream factors in the disease pathway, eliminating the possibility of reverse causation. To ensure the reliability of our MR analyses and mitigate potential pleiotropic effects, we incorporated multiple MR approaches, containing MR-PRESSO, Weighted mode, Weighted median, and MR-Egger tests.

This bidirectional MR examination showed a correlation between elevated genetically influenced CRP and IL-8 levels and a heightened likelihood of IgAV. In contrast, our findings indicated a causal connection between IgAV and reduced TNF-β concentrations, alluding to a potential proinflammatory influence of IgAV. Nevertheless, no finding was observed to support a causal link between PCT or other inflammatory cytokines and IgAV.

Our findings validate the assumption that circulating inflammatory regulators exert significant roles in facilitating the progression of IgAV and function as prognostic indicators correlating with the stage of IgAV. The deposition of IgA in the vascular endothelium is the fundamental characteristic of IgAV disease. However, the relationship between IgA deposition and circulating inflammatory factors is more fluid but somewhat reciprocal. On the one hand, the IgA immune complexes formed in IgAV bind to FcαRI, which activates downstream ITAM signaling, resulting in the activation of immune cells, characterized by neutrophil migration and subsequent abnormal release of inflammatory mediators (47). On the other hand, circulating inflammatory factors, such as G-CSF and VEGF, influence the activation and migration of inflammatory cells by regulating the expression of FcαRI or altering vascular permeability (48, 49).

Activated inflammatory cells release IL-6, an essential candidate in the proliferation of galactose-deficient IgA1, ultimately leading to further increases in IgA immune complexes and the progression of IgAV (50). As previously reported, aberrant IL-6 decreases the transcription of C1GALT1 by activating the JAK/STAT pathway, thereby inducing the generation of galactose-deficient IgA1 (51), a part of the pathogenesis of IgAV. Although our study’s magnitude of IgAV on IL-6 was positive, we did not find a significant correlation. Therefore, the previously reported positive association requires further clarification. Similarly, VEGF, a critical circulating inflammatory factor regulating vascular permeability, is considered a crucial contributor to the progress of IgAV. Studies have detected a significant increase in VEGF levels during the acute phase of IgAV patients but no significant difference compared to healthy individuals during the resolution phase (49). These may explain why, in our study, despite the elevated trend of VEGF exposure among IgAV patients, it did not reach statistical significance. Previous studies on the relationship between IgAV and circulating inflammatory factors have yielded inconsistent results, and the paradoxical biofunction of IL-8 gene polymorphisms should be clarified (18, 19). Therefore, we employed genetic variants as instrumental variables to mitigate the impact of variations in inflammatory cytokine levels resulting from medical interventions or complications associated with the IgAV.

Currently, we identified CRP and IL-8 as risk factors for IgAV, while inversely, our findings also revealed that IgAV was causally implicated with increased TNF-β concentrations. CRP, as an acute-phase reactant, directly activates endothelial cells, promoting the release of inflammatory mediators and adhesion molecules. Indirectly, CRP activates the immune system, leading to inflammation and subsequent activation of inflammatory cells (52). These processes collectively contribute to developing IgA vasculitis by initiating and amplifying the inflammatory response within the vascular wall. Recent studies have consistently demonstrated a significant association between disease progression in IgAV and increased CRP levels (53, 54). Interestingly, a study investigated IgAV patients with MEFV mutations and found that those without genetic mutations significantly increased CRP levels, which correlated significantly with increased serum IgA concentrations. This observation suggested the existence of novel factors linking elevated CRP levels to increased serum IgA (55). However, the relationship between CRP and the progression of renal disease in IgAV patients remains controversial. A study has indicated higher CRP levels in IgAV patients with renal impairment than those without renal disease (56). In contrast, another study has suggested no correlation between CRP and the histopathological grading of renal disease in IgAV patients (57). Future research is needed to reveal the causal relationship between CRP and kidney subtypes in patients with IgAV.

IL-8, a chemotactic and inflammatory cytokine, attracts and activates neutrophils. Levels of IL-8 in supernatants from children with active IgAV were higher than those of healthy individuals, reflecting the function of IL-8 in the mechanisms of perivascular neutrophil infiltration (58). This vascular damage further enhances the recruitment of immune cells, leading to increased production of IgA antibodies (15). Studies have indicated a potent elevation of IL-8 in IgAV patients (10), and it could serve as a prognostic biomarker for IgAV nephritis (59). Dapsone showed a therapeutic effect in IgAV by blocking the action of IL-8 (60). Additionally, we were surprised to observe the negative causal interaction between IgAV and TNF-β. T-lymphocytes predominantly express the TNF-β and act as a pro-inflammatory factor like TNF-α (61). However, when the course of IgAV entered the remission stage, the follicular helper T cells were decreased in children with active IgA vasculitis cells, which may lead to decreased TNF-β level (62). A previous study that included 152 cases found that experimental results of TNF were significantly reduced in IgAV patients with or without renal involvement (63). We speculate that this result may indirectly support our findings that the disease course of IgAV is negatively related to the TNF-β level. As known, there are limited and conflicting reports on the role of TNF in IgAV. Therefore, future study with expanded sample sizes and more precise methods is demanded to elucidate the actual role of TNF in IgAV.

The findings of our study suggest that inflammatory cytokines play critical roles in the pathogenesis of IgAV, which may serve as potential targets for IgAV prevention and treatment, and the underlying mechanisms might be involved in complex interactions or synergies with environmental or infectious factors related to IgAV. For instance, streptococcal infection, the most common infectious trigger for childhood IgAV, may induce or enhance the production of CRP and IL-8 by activating the immune system and endothelial cells, thereby leading to complement system activation and inflammation (64–66). Conversely, eliminating streptococcal infection may reduce the relapse/recurrence of IgAV by decreasing CRP and IL-8 levels (64). Another factor is HSPs, which protect other proteins under stress and have antiviral effects by interacting with immune pathways (67). HSP60 riggers the secretion of IL-8 through binding to toll-like receptors (TLRs) on monocytes (68), whereas HSP90 contributes to IL-8 elevation through regulating TLR-4 and further leads to the inflammation process (69). Collectively, HSPs may modulate the inflammatory response in IgAV by regulating cytokine levels. Moreover, viral infection, another possible trigger for IgAV, may also affect cytokine levels by hijacking some HSPs to facilitate viral invasion, replication, and maturation (70). As well, viral infection is a potential candidate for inducing cytokine storm, a hyper-inflammatory condition that can contribute to tissue damage and organ failure in severe cases of IgAV (71). Therefore, understanding the interaction or synergistic mechanisms of environmental or infectious factors and cytokines in IgAV may provide new insights into the etiology and pathophysiology of IgAV.

Our research possesses multiple benefits. The robust genetic instrumental variable primarily enabled us to conduct the first comprehensive MR analysis concerning the relationship between inflammatory cytokines and IgAV risk. This groundbreaking attempt is significant in determining if a genetic predisposition to IgAV can alter circulating inflammatory factors. Additionally, we explore the impact of high or low circulating inflammatory factors on IgAV risk. These aspects need to be addressed in prior MR studies. Moreover, the genetic instruments of our study abide by the STROBE-MR Statement, making them independent of confounders within an exposure-outcome relationship and adjusted for genetic principal genetic components to minimize collider bias, which may potentially infringe upon fundamental MR hypothesis (22). Lastly, we employed a less stringent cut-off (1 x 10^-5^), compared to the standard threshold of 5 x 10^-8^, for lower frequency variants in European ancestry to expand possibilities (72).

There are several limitations in our current study. Firstly, inherent limitations in MR studies should be considered. MR analysis can only use existing genetic data. It cannot cover any other non-genetic factors that may affect the occurrence and development of IgAV, such as demographics and lifestyle. Secondly, our study should have extensively addressed the subgroup complexities (e.g., renal or gastrointestinal involvement) associated with IgAV, including its varied etiology and prognostic heterogeneity. It is desirable to explore these complications, which can be constrained by insufficient statistical capacity and limited sample size. Thirdly, using a less stringent threshold of 1 x 10^-5^ to collect more SNPs for PCT, circulating inflammatory factors, and IgAV may increase the risk of false positives and reduce the power of MR analysis. Future studies with larger sample sizes, more comprehensive genetic data, and robust instruments will be designed to verify our present conclusion. Additionally, alterations in circulating inflammatory factors could be affected by unpredictable variables present in real-life clinical settings. For instance, the potential bias resulting from misclassification in the FinnGen biobank may impact the results, as patients lost to follow-up before developing purpura (such as those who emigrated from Finland) lacked medical records. Moreover, considering the limited sample size in our validation cohort, studies with large samples are expected to validate our research further. Furthermore, although previous studies do not support the impact of gender on the susceptibility and prognosis of IgAV, the incidence of IgAV is slightly higher in males than females (73). Nonetheless, significantly more females are enrolled in the FinnGen Biobank, potentially leading to the oversight of specific associations. Similarly, even though MR allows for assessing the lifelong effects of genetically predicted inflammatory cytokines, it may not directly capture the suppression of these factors in adult life due to the influence of various unreported regulators. We mitigated the biases by excluding SNPs related to survival proxied by age at recruitment, and future studies should consider more inflammatory factors to conduct a more comprehensive and in-depth analysis. Lastly, it is essential to acknowledge that our findings primarily apply to individuals of European ethnicity, as access to other racial populations was limited. However, it is crucial to recognize that race-related issues frequently lead to treatment underutilization and unplanned interruptions in IgAV management.

## 5 Conclusions

Our MR study demonstrates a causal correlation between inflammation and IgAV. We highlight the role of two key circulating inflammatory factors, CRP and IL-8, in driving the causal effects on IgAV. Moreover, it is worth investigating whether TNF-β levels are reduced in individuals susceptible to IgAV, which may affect prognosis. Further research is warranted to validate these findings thoroughly.

## Data availability statement

The original contributions presented in the study are included in the article/**Supplementary Material**. Further inquiries can be directed to the corresponding author.

## Ethics statement

Ethical approval was not required for the study involving humans in accordance with the local legislation and institutional requirements. Written informed consent to participate in this study was not required from the participants or the participants’ legal guardians/next of kin in accordance with the national legislation and the institutional requirements.

## Author contributions

Conceptualization: JQ and CJ; Data collection: JQ, LZ, BK and TL; Formal analysis: BK, TL and CK; Methodology: JQ and LZ; Validation: JQ, LZ, CK and CJ; Visualization: JQ and LZ; Writing original draft: JQ and LZ; Writing-review and editing: JQ and CJ; All authors contributed to the article and approved the submitted version.

## Glossary

**Table:**

IgAV	IgA vasculitis
HSPs	Heat shock proteins
TLRs	toll-like receptors
CRP	C-reactive protein
PCT	procalcitonin
GS	genome-wide significant
GWAS	genome-wide association study
IVW method	inverse-variance weighted method
MR-PRESSO	MR-pleiotropy residual sum and outlier
OR	Estimate odds ratio
CI	confidence interval
BMI	Body mass index
bNGF	beta nerve growth factor
CTACK	cutaneous T-cell attracting chemokine
FGFBasic	basic fibroblast growth factor
GCSF	granulocyte colony-stimulating factor
GROa	growth-regulated oncogene-a
HGF	hepatocyte growth factor
IFNg	interferon gamma
IL	interleukin
IP	interferon-gamma-induced protein
MCP1	monocyte chemotactic protein-1
MCP3	monocyte-specific chemokine 3
MCSF	macrophage colony- stimulating factor
MIF	macrophage-migration inhibitory factor
MIG	monokine induced by interferon gamma
MIP1a	macrophage inflammatory protein-1a
MIP1b	macrophage inflammatory protein-1b
PDGFbb	platelet-derived growth factor BB
RANTES	regulated on Activation, Normal T Cell Expressed and Secreted
SCF	stem cell factor
SNPs	single-nucleotide polymorphisms
TRAIL	TNF-related apoptosis-inducing ligand
VEGF	vascular endothelial growth factor
