# Supplementary material for: Causal relationships between circulating inflammatory factors and IgA vasculitis: a bidirectional Mendelian randomization study
Source: Front Immunol. 2023 Sep 11;14:1248325. doi: 10.3389/fimmu.2023.1248325 (PMC10518517; doi:10.3389/fimmu.2023.1248325)
Supplement: Supplementary file 1 [file DataSheet_1.pdf]

Causal relationships between circulating inflammatory factors and IgA vasculitis: A bidirectional Mendelian randomization study

Jiading Qin, Ling Zhang, Bo Ke, Tingting Liu, Chunfang Kong
and Chenghao Jin

Supplementary materials

Figure of contents

Figure S1. The causal effects and sensitivity analysis of CRP on IgAV in validation cohort. A) The causal effect of CRP on IgAV. B) MR leave-one out sensitivity analysis for CRP on IgAV. C) MR effect size for CRP on IgAV. D) Causal estimate for different MR tests.

Table of contents

Table S1. Details of C-reactive protein predicting SNPs with IgA vasculitis.

Table S2. Details of IgA vasculitis predicting SNPs with C-reactive protein.

Table S3. Bidirectional causal association between IgA vasculitis and C-reactive protein using Mendelian randomization.

Table S4. Details of C-reactive protein predicting SNPs with IgA vasculitis in validation cohort.

Table S5. Details of procalcitonin predicting SNPs with IgA vasculitis.

Table S6. Details of IgA vasculitis predicting SNPs with procalcitonin.

Table S7. Bidirectional causal association between IgA vasculitis and procalcitonin using Mendelian randomization.

Table S8. Details of circulating inflammatory regulators predicting SNPs with IgA vasculitis (with genome-wide significant SNPs).

Table S9. Details of circulating inflammatory regulators predicting SNPs with IgA vasculitis (with SNPs reaching P < 1 x 10-5).

Table S10. Association of circulating inflammatory regulators with IgA vasculitis using Mendelian randomization (with genome-wide significant SNPs).

Table S11. Association of circulating inflammatory regulators with IgA vasculitis using Mendelian randomization (with SNPs reaching P< 1 x 10-5).

Table S12. Details of IgA vasculitis predicting SNPs with circulating inflammatory regulators (with SNPs reaching P<1 x 10-5).

Table S13. Association of IgA vasculitis with circulating inflammatory regulators using Mendelian randomization (with SNPs reaching P<1 x 10-5).

Figure S1

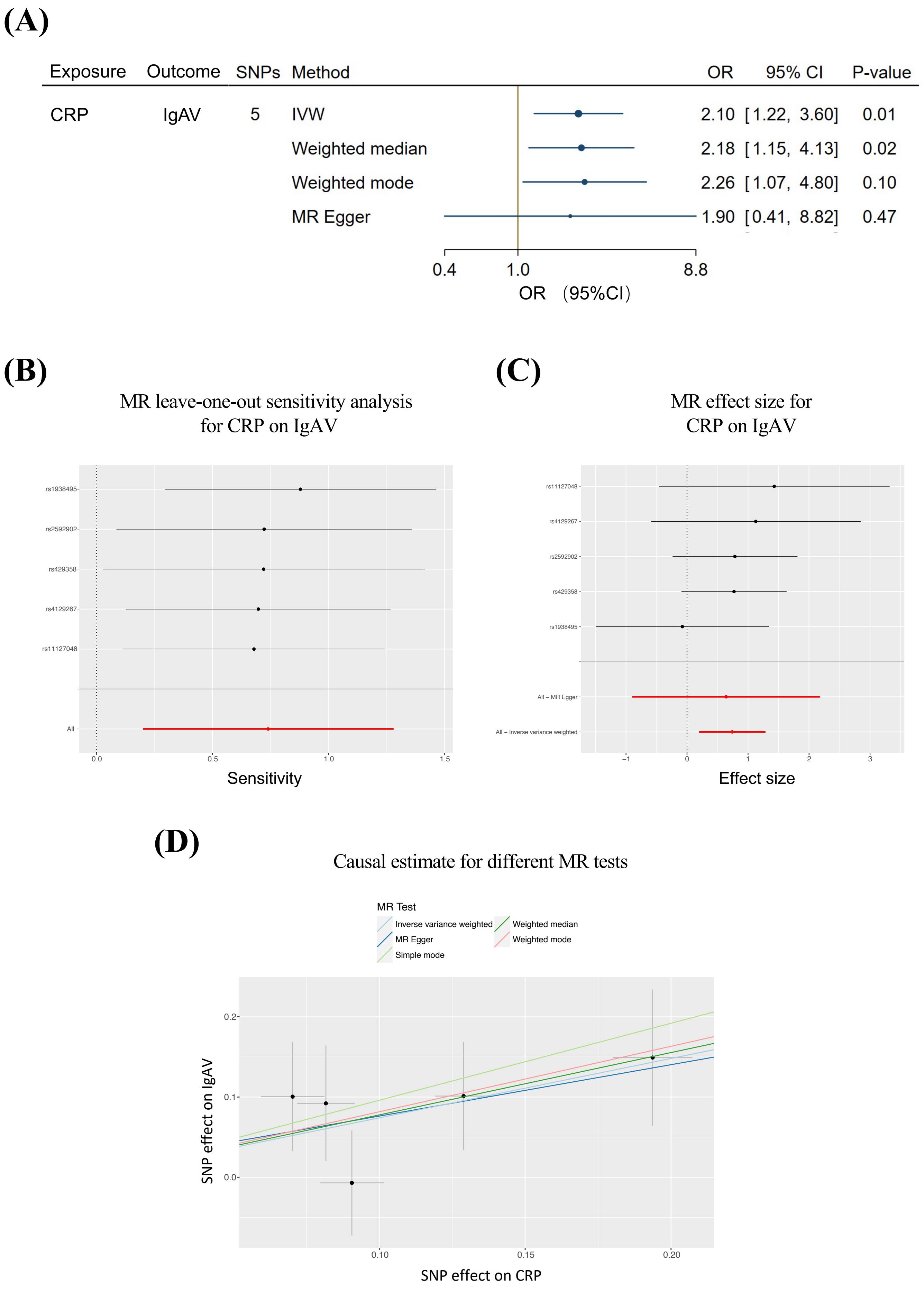
(A)
Exposure
Outcome
SNPs
Method
OR
95% CI
P-value
CRP
IgAV
5
IVW
2.10 [1.22, 3.60]
0.01
Weighted median
2.18 [1.15, 4.13]
0.02
Weighted mode
2.26 [1.07, 4.80]
0.10
MR Egger
1.90 [0.41, 8.82]
0.47
0.4
1.0
8.8
OR (95%CI)
(B)
MR leave-one-out sensitivity analysis
for CRP on IgAV
rs1938495
rs2592902
rs429358
rs4129267
rs11127048
All
0.0
0.5
1.0
1.5
Sensitivity
(C)
MR effect size for
CRP on IgAV
rs11127048
rs4129267
rs2592902
rs429358
rs1938495
All – MR Egger
All – Inverse variance weighted
−1
0
1
2
3
Effect size
(D)
Causal estimate for different MR tests
MR Test
Inverse variance weighted
MR Egger
Simple mode
Weighted median
Weighted mode
0.2
0.1
0.0
SNP effect on IgAV
0.10
0.15
0.20
SNP effect on CRP

Table S1. Details of C-reactive protein predicting SNPs with IgA vasculitis.

	C-reactive protein					IgA vasculitis		*F*-statistics
SNP	Effect allele	Other allele	*P*-value	Beta	Standard Error	log(OR)	Standard Error	
rs10512597	C	T	4.44E-14	0.037	0.005	-0.010	0.078	88.252
rs1051338	G	T	2.27E-09	0.024	0.004	0.010	0.069	52.672
rs10521222	T	C	2.06E-22	-0.104	0.011	-0.250	0.207	204.533
rs10832027	A	G	4.43E-12	0.026	0.004	-0.086	0.066	64.596
rs10925027	C	T	4.25E-21	-0.036	0.004	-0.025	0.067	135.108
rs12202641	T	C	3.00E-10	-0.023	0.004	0.035	0.067	53.812
rs12587622	A	G	8.52E-09	-0.021	0.004	-0.055	0.065	46.849
rs1260326	C	T	2.72E-92	-0.073	0.004	-0.101	0.069	558.969
rs12960928	C	T	1.91E-09	0.024	0.004	0.063	0.082	48.838
rs12995480	C	T	1.24E-10	0.031	0.005	0.091	0.089	59.482
rs13233571	T	C	2.95E-25	-0.057	0.005	0.023	0.098	147.743
rs13409371	A	G	5.07E-36	0.048	0.004	0.117	0.072	247.227
rs1441169	G	A	2.27E-11	-0.025	0.004	-0.052	0.067	67.012
rs1490384	T	C	2.65E-12	-0.025	0.004	-0.006	0.065	66.665
rs1509394	T	C	6.05E-10	0.026	0.004	-0.043	0.066	70.933
rs1582763	A	G	2.37E-09	-0.022	0.004	-0.059	0.073	49.453
rs17658229	C	T	5.50E-09	0.056	0.010	-0.177	0.153	58.563
rs178810	T	C	2.95E-08	0.020	0.004	-0.019	0.067	42.678
rs1800961	T	C	4.63E-23	-0.112	0.011	0.059	0.158	176.079
rs1805096	A	G	2.17E-183	-0.104	0.004	0.017	0.066	1124.539
rs1880241	G	A	8.41E-14	-0.028	0.004	-0.082	0.067	82.045
rs2064009	T	C	2.28E-14	0.027	0.004	-0.002	0.066	77.505
rs2239222	G	A	9.87E-20	0.035	0.004	-0.057	0.068	126.260
rs2315008	G	T	5.36E-10	0.023	0.004	-0.043	0.074	51.237
rs2352975	C	T	6.43E-10	0.025	0.004	0.063	0.075	56.777
rs2710804	C	T	1.30E-08	0.021	0.004	-0.037	0.068	45.519
rs2794520	T	C	1.00E-200	-0.182	0.004	-0.125	0.068	3245.113
rs2836878	A	G	7.71E-26	-0.043	0.004	-0.159	0.076	158.767
rs2852151	A	G	1.36E-11	0.025	0.004	-0.087	0.066	63.853
rs2891677	T	C	1.59E-08	0.020	0.004	0.016	0.066	42.454
rs3122633	C	T	1.68E-12	0.027	0.004	-0.064	0.069	68.926
rs3134899	T	C	4.93E-08	0.023	0.004	-0.129	0.078	42.129
rs340005	A	G	1.01E-15	0.030	0.004	-0.064	0.073	91.858
rs387976	C	A	1.05E-10	0.026	0.004	-0.033	0.072	62.509
rs4092465	G	A	3.11E-10	0.027	0.004	-0.060	0.068	74.384
rs4129267	T	C	1.20E-129	-0.088	0.004	-0.092	0.072	788.792
rs4246598	A	C	5.11E-10	0.022	0.004	-0.119	0.067	52.427
rs4420638	G	A	1.00E-200	-0.229	0.006	-0.163	0.074	3413.454

rs4655802	A	G	1.88E-09	-0.025	0.004	0.019	0.067	64.191
rs4656849	G	A	4.91E-54	0.058	0.004	-0.143	0.071	339.288
rs469772	T	C	5.54E-12	-0.031	0.005	0.111	0.080	66.366
rs4767920	A	G	4.00E-15	-0.039	0.005	-0.123	0.091	85.473
rs4841132	G	A	2.00E-25	0.065	0.006	0.058	0.098	151.890
rs6001193	G	A	6.53E-14	-0.028	0.004	0.051	0.068	76.295
rs644234	G	T	1.13E-09	0.023	0.004	0.013	0.066	51.521
rs6601302	G	T	9.80E-12	-0.031	0.004	-0.053	0.085	78.233
rs6672627	A	C	2.89E-13	-0.037	0.005	-0.028	0.085	74.561
rs7121935	A	G	5.28E-09	-0.022	0.004	-0.074	0.069	48.559
rs7310409	G	A	1.00E-200	0.137	0.004	-0.068	0.067	1957.546
rs9271608	G	A	2.33E-17	0.042	0.005	0.092	0.092	129.304
rs9284725	A	C	7.34E-11	-0.027	0.004	-0.005	0.098	58.736

Abbreviations: log(OR), log odds ratio.

F-statistic >10 suggests sufficient strength to ensure the validity of the instrumental variable.

Beta for CRP represents change in percent per 1copy of effect allele

Log(OR) for IgA vasculitis represents log(OR) change in IgA vasculitis risk per 1 copy of effect allele.

Table S2. Details of IgA vasculitis predicting SNPs with C-reactive protein.

IgA vasculitis						C-reactive protein		*F*-statistics
SNP	Effect allele	Other allele	*P*-value	log(OR)	Standard Error	Beta	Standard Error	
rs2032842	A	G	2.07E-06	-0.380	0.080	0.010	0.005	11416.822
rs2211860	T	C	6.37E-06	0.298	0.066	-0.018	0.004	9986.565
rs6462017	T	C	4.37E-06	-0.306	0.067	0.005	0.004	10393.069
rs9531785	C	T	2.59E-06	-0.327	0.070	-0.007	0.004	10880.259

Abbreviations: log(OR), log odds ratio.

F-statistic >10 suggests sufficient strength to ensure the validity of the instrumental variable.

Beta for CRP represents change in percent per 1 copy of effect allele.

Log(OR) for IgA vasculitis represents log(OR) change in IgA vasculitis risk per 1 copy of effect allele.

Table S3. Bidirectional causal association between IgA vasculitis and C-reactive protein using Mendelian randomization.

		Inverse variance weighted							MR-Egger					Weighted Median	Weighted Mode	MR-PRESSO
Exposure	Outcome	No. of SNPs	OR/ Beta	95% CI	*P*-val	Q	Q *P*-val	I^2	OR/ Beta	95% CI	*P*-val	intercept	intercept *P*-val	*P*-val	*P*-val	Global Test *P*-val
CRP*	IgAV	51	1.412	1.010 - 1.975	0.044	50.647	0.448	1.3%	1.867	1.154 - 3.023	0.014	-0.024	0.122	0.008	0.010	0.471
IgAV	CRP	4	-0.018	-0.052 - 0.017	0.313	23.683	0.000	87.3%	0.083	-0.381 - 0.548	0.758	-0.033	0.711	0.103	0.411	0.008

Abbreviations: CI, Confidence interval; CRP, C-reactive protein; IgAV, IgA vasculitis; OR, odds ratio; P-val, P-value; Q, Cochran Q statistics; SNPs, single nucleotide polymorphisms.

* represents the SNPs associated with the exposure at a genome-wide significant level ($P<5\times10^{-8}$).

OR and 95% CI represent change in odds ratio of IgA vasculitis per 1 percent increase in CRP level.

Beta and 95% CI represent change in percent of CRP per log odds increase in IgA vasculitis.

Table S4. Details of C-reactive protein predicting SNPs with IgA vasculitis in validation cohort.

C-reactive protein						IgA vasculitis		
SNP	Effect allele	Other allele	*P*-val	Beta	Standard Error	log(OR)	Standard Error	F-statistics
rs11127048	A	G	7.81E-11	-0.070	0.011	-0.101	0.068	502.163
rs1938495	G	A	1.60E-16	-0.091	0.011	0.007	0.066	890.613
rs2592902	T	G	2.51E-39	-0.129	0.010	-0.101	0.067	1725.394
rs4129267	T	C	7.42E-17	-0.082	0.010	-0.092	0.072	605.445
rs429358	C	T	7.27E-46	-0.194	0.014	-0.149	0.085	2453.062

Abbreviations: log(OR), log odds ratio.

F-statistic >10 suggests sufficient strength to ensure the validity of the instrumental variable.

Beta for CRP represents change in percent per 1copy of effect allele

Log(OR) for IgA vasculitis represents log(OR) change in IgA vasculitis risk per 1 copy of effect allele.

Table S5. Details of procalcitonin predicting SNPs with IgA vasculitis.

Procalcitonin						IgA vasculitis		*F*-statistics
SNP	Effect allele	Other allele	*P*-value	Beta	Standard Error	log(OR)	Standard Error	
rs112431269	T	C	6.76E-06	-0.419	0.093	0.095	0.232	1592.439
rs113712971	T	C	4.27E-06	-0.233	0.051	0.116	0.130	1621.956
rs114802537	A	G	2.51E-06	0.549	0.117	-1.042	1.028	1553.318
rs12043613	G	A	2.34E-06	0.212	0.045	-0.032	0.135	1518.520
rs13288876	A	G	3.31E-06	-0.228	0.049	-0.056	0.135	1607.827
rs141164217	C	A	4.27E-06	0.649	0.141	-0.108	0.206	1615.323
rs1446528	G	A	9.12E-07	0.138	0.028	0.036	0.077	1600.328
rs145214525	A	G	1.12E-06	-0.588	0.121	0.392	0.533	1879.734
rs147828190	T	C	3.98E-08	0.443	0.081	0.096	0.150	2210.099
rs186735186	G	A	5.01E-06	0.467	0.102	-0.440	0.732	1433.179
rs2270263	T	C	3.16E-07	0.135	0.026	0.126	0.067	1916.976
rs2427459	T	C	3.16E-06	0.117	0.025	0.033	0.066	1482.173
rs4416082	C	T	8.13E-06	0.125	0.028	0.055	0.074	1316.132
rs57626295	G	T	2.75E-06	-0.132	0.028	-0.077	0.071	1475.148
rs61986621	T	C	3.80E-06	0.145	0.031	-0.010	0.071	1515.556
rs719070	A	G	6.31E-06	0.152	0.034	-0.110	0.091	1354.107
rs74093831	A	C	1.15E-07	0.149	0.028	0.002	0.072	1943.340
rs76051678	A	G	3.98E-06	-0.232	0.050	-0.211	0.110	1566.276
rs78121206	A	G	8.13E-06	-0.355	0.080	0.412	0.242	1348.917
rs78816719	C	T	4.68E-06	0.307	0.067	-0.310	0.267	1414.830
rs78832381	T	C	8.71E-06	0.287	0.065	-0.098	0.366	1354.144
rs79121245	A	G	6.76E-06	0.419	0.093	0.180	0.293	1389.601
rs9474592	C	A	6.31E-07	-0.154	0.031	0.006	0.084	1771.069

Abbreviations: log(OR), log odds ratio.

F-statistic >10 suggests sufficient strength to ensure the validity of the instrumental variable.

Beta for procalcitonin represents change in percent per 1 copy of effect allele.

Log(OR) for IgA vasculitis represents log(OR) change in IgA vasculitis risk per 1 copy of effect allele.

Table S6. Details of IgA vasculitis predicting SNPs with procalcitonin.

IgA vasculitis						Procalcitonin		*F*-statistics
SNP	Effect allele	Other allele	*P*-value	log(OR)	Standard Error	Beta	Standard Error	
rs111407737	G	T	3.84E-06	1.068	0.231	-0.022	0.084	12749.218
rs115469485	G	A	3.94E-06	0.933	0.202	-0.024	0.075	12357.221
rs142409457	C	T	6.81E-06	0.790	0.176	0.140	0.066	11616.037
rs150507973	A	G	2.92E-07	0.694	0.135	-0.060	0.057	14832.719
rs2032842	A	G	2.07E-06	-0.380	0.080	0.012	0.033	11416.822
rs2211860	T	C	6.37E-06	0.298	0.066	-0.026	0.026	9986.565
rs4239724	G	A	9.50E-06	0.373	0.084	-0.018	0.031	9759.864
rs4670545	A	G	5.22E-06	0.324	0.071	-0.010	0.025	10176.533
rs4863841	G	T	2.05E-07	-0.373	0.072	0.017	0.029	13582.110
rs58327154	T	C	1.02E-06	0.505	0.103	0.043	0.041	12751.648
rs6462017	T	C	4.37E-06	-0.306	0.067	0.033	0.025	10393.069
rs7009028	C	T	2.88E-06	-0.812	0.174	-0.015	0.071	12465.322
rs72711557	A	G	7.60E-06	0.487	0.109	0.051	0.041	10259.341
rs72744256	A	G	6.45E-06	0.617	0.137	-0.056	0.047	10903.625
rs76945286	A	G	4.19E-06	1.320	0.287	-0.005	0.120	13311.131
rs78230571	A	G	3.12E-07	3.727	0.729	-0.098	0.113	23950.911
rs884835	G	A	1.82E-06	0.483	0.101	0.052	0.036	11906.262
rs9531785	C	T	2.59E-06	-0.327	0.070	0.002	0.026	10880.259

Abbreviations: log(OR), log odds ratio.

F-statistic >10 suggests sufficient strength to ensure the validity of the instrumental variable.

Beta for procalcitonin represents change in percent per 1 copy of effect allele.

Log(OR) for IgA vasculitis represents log(OR) change in IgA vasculitis risk per 1 copy of effect allele.

Table S7. Bidirectional causal association between IgA vasculitis and procalcitonin using Mendelian randomization.

		Inverse variance weighted							MR-Egger					Weighted Median	Weighted Mode	MR-PRESSO
Exposure	Outcome	No. of SNPs	OR/ Beta	95% CI	*P*-val	Q	Q *P*-val	I^2	OR/ Beta	95% CI	*P*-val	intercept	intercept *P*-val	*P*-val	*P*-val	Global Test *P*-val
procalcitonin	IgAV	23	1.051	0.837 - 1.320	0.665	19.131	0.637	0.0%	0.738	0.447 - 1.218	0.247	0.076	0.135	0.986	0.834	0.636
IgAV	procalcitonin	18	-0.012	-0.044 - 0.021	0.475	15.841	0.535	0.0%	-0.003	-0.060 - 0.053	0.913	-0.006	0.718	0.307	0.335	0.573

Abbreviations: CI, Confidence interval; IgAV, IgA vasculitis; OR, odds ratio; P-val, P-value; Q, Cochran Q statistics; SNPs, single nucleotide polymorphisms.

OR and 95% CI represent change in odds ratio of IgA vasculitis per 1 percent increase in procalcitonin level.

Beta and 95% CI represent change in percent of procalcitonin per log odds increase in IgA vasculitis.

Table S8. Details of circulating inflammatory regulators predicting SNPs with IgA vasculitis (with genome-wide significant SNPs).

Circulating inflammatory regulators						IgA vasculitis		*F*-statistics
SNP	Effect allele	Other allele	*P*-value	Beta	Standard Error	log(OR)	Standard Error	
**MIP1b**								
rs113010081	C	T	3.85E-140	0.595	0.024	0.033	0.098	53.192
rs113877493	T	C	1.62E-173	-0.612	0.022	-0.068	0.089	277.097
rs117453826	G	A	5.07E-22	0.577	0.059	0.205	0.222	404.228
rs141102180	T	G	1.08E-16	0.323	0.039	-0.216	0.169	781.267
rs17641689	G	A	1.28E-16	0.245	0.029	0.156	0.105	1058.876
rs2079664	G	A	1.51E-08	-0.100	0.018	0.015	0.074	17.858
**Eotaxin**								
rs112347425	T	C	8.65E-09	0.158	0.028	-0.063	0.115	902.319
rs12075	A	G	1.33E-26	0.167	0.016	0.004	0.066	3054.623
rs2024050	G	A	1.10E-08	-0.173	0.030	0.026	0.127	873.191
rs2228467	C	T	2.27E-46	0.416	0.029	-0.089	0.123	5436.932
**MCP1**								
rs12075	A	G	1.44E-44	0.219	0.016	0.004	0.066	5275.670
rs2036297	G	A	1.09E-13	-0.119	0.016	0.103	0.068	1451.474
rs2288370	C	T	2.25E-10	0.103	0.016	-0.011	0.068	1088.263
rs7632755	A	G	1.18E-20	0.294	0.032	-0.089	0.126	2575.962
**PDGFbb**								
rs13412535	A	G	2.46E-55	0.335	0.021	0.038	0.083	8245.312
rs2324229	C	T	3.48E-08	-0.089	0.016	-0.045	0.068	812.821
rs4965869	T	C	5.66E-24	0.184	0.018	-0.019	0.076	2751.509
rs55680718	T	C	1.86E-08	-0.138	0.025	0.036	0.101	877.757
**SCGFb**								
rs116924815	T	C	1.74E-16	0.608	0.074	-0.085	0.199	4382.700
rs117716477	A	C	1.34E-23	0.838	0.084	0.205	0.259	5055.983
rs17876031	G	A	2.25E-09	0.151	0.026	-0.037	0.070	2197.656
rs181218758	C	T	5.58E-11	0.383	0.059	0.043	0.178	2233.572
rs4656185	A	G	1.16E-15	0.205	0.026	0.011	0.071	3918.118
**VEGF**								
rs13209117	A	G	5.28E-11	0.130	0.020	0.046	0.076	1404.664
rs34467391	AG	A	3.40E-14	0.129	0.017	0.004	0.066	1811.600
rs6921438	A	G	2.09E-171	-0.490	0.018	0.078	0.066	29513.584
**IL-18**								
rs115267715	T	C	1.72E-08	0.451	0.080	-0.221	0.235	1758.826
rs17229943	C	A	1.62E-11	0.312	0.046	-0.201	0.109	3932.081
rs385076	C	T	1.66E-22	0.243	0.025	0.000	0.069	6027.676
rs71478720	T	C	3.71E-22	-0.267	0.028	0.069	0.076	5810.045
**IL-12p70**								

rs34467391	AG	A	6.78E-11	0.103	0.016	0.004	0.066	1137.147
rs4349809	G	T	2.56E-124	-0.378	0.016	0.070	0.066	16589.134
rs7088799	G	T	6.29E-10	0.100	0.016	0.020	0.067	1025.592
**IL-16**								
rs1801020	G	A	4.53E-10	-0.173	0.027	-0.030	0.074	2535.882
rs4253283	C	T	1.75E-08	-0.146	0.026	0.084	0.071	1967.945
rs4778636	A	G	1.11E-30	-0.727	0.063	-0.087	0.159	10115.378
**TRAIL**								
rs138987090	G	A	4.50E-23	0.750	0.075	0.065	0.281	3420.778
rs193112415	C	T	2.15E-62	1.042	0.062	0.318	0.240	9255.143
rs57396456	C	T	1.25E-27	0.563	0.052	0.014	0.218	3107.620
rs62093514	T	C	6.86E-82	1.062	0.055	0.101	0.225	11393.437
rs74778900	T	C	2.59E-28	0.591	0.053	-0.351	0.289	2005.462
rs79287178	A	G	9.12E-25	-0.432	0.042	0.319	0.166	3269.135

Abbreviations: log(OR), log odds ratio.

F-statistic >10 suggests sufficient strength to ensure the validity of the instrumental variable.

Beta for circulating inflammatory regulators represents change in standard deviation per 1 copy of effect allele.

Log(OR) for IgA vasculitis represents log(OR) change in IgA vasculitis risk per 1 copy of effect allele.

Table S9. Details of circulating inflammatory regulators predicting SNPs with IgA vasculitis (with SNPs reaching P < 1 x 10-5).

Table S9 displayed in Table S9.xlsx.

Table S10. Association of circulating inflammatory regulators with IgA vasculitis using Mendelian randomization (with genome-wide significant SNPs).

		Inverse variance weighted						MR-Egger					Weighted Median	Weighted Mode	MR-PRESSO
Exposure	No. of SNPs	OR	95% CI	*P*-val	Q	Q *P*-val	I^2	OR	95% CI	*P*-val	intercept	intercept *P*-val	*P*-val	*P*-val	Global Test *P*-val
**Chemokines**															
MIP1b	6	1.109	0.913 - 1.346	0.297	4.349	0.500	0.0%	1.119	0.763-1.640	0.596	-0.005	0.959	0.410	0.436	0.572
Eotaxin	4	0.857	0.562 - 1.306	0.474	0.355	0.949	0.0%	0.751	0.2640 - 2.1354	0.645	0.032	0.811	0.450	0.542	0.943
MCP1	4	0.819	0.539 - 1.244	0.349	1.964	0.580	0.0%	1.131	0.365 - 3.506	0.851	-0.060	0.608	0.747	0.915	0.579
**Growth Factors**															
PDGFbb	4	1.065	0.725 - 1.563	0.749	0.736	0.865	0.0%	1.019	0.442 - 2.352	0.969	0.010	0.919	0.794	0.792	0.899
SCGFb	5	1.029	0.747 - 1.418	0.861	1.137	0.888	0.0%	1.175	0.655 - 2.110	0.626	-0.045	0.632	0.768	0.694	0.894
VEGF	3	0.884	0.484 - 1.186	0.439	0.838	0.658	0.0%	0.758	0.484 - 1.186	0.439	0.058	0.567	0.278	0.373	#
**Interleukins**															
IL-18	4	0.757	0.548 - 1.047	0.092	2.235	0.525	0.0%	0.219	0.028 - 1.744	0.288	0.344	0.357	0.234	0.549	0.550
IL-12p70	3	0.862	0.626 - 1.186	0.363	0.394	0.821	0.0%	0.744	0.420 - 1.320	0.497	0.042	0.653	0.348	0.426	#
IL-16	3	1.034	0.725 - 1.473	0.854	1.812	0.404	0.0%	1.257	0.691 - 2.289	0.591	-0.067	0.565	0.527	0.609	#
**Other**															
TRAIL	6	1.019	0.763 - 1.359	0.901	7.139	0.211	30.0%	2.201	1.091 - 4.438	0.092	-0.610	0.084	0.543	0.464	0.253

Abbreviations: CI, Confidence interval; OR, Odds Ratio; P-val, P-value; Q, Cochran Q statistics; SNPs, single nucleotide polymorphisms.

#Not enough SNPs for MR-PRESSO analysis.

OR and 95% CI represent change in odds ratio of IgA vasculitis per 1 SD increase in circulating inflammatory regulators level.

After correcting for multiple comparison, P-value < 0.05 was considered as significant.

Table S11. Association of circulating inflammatory regulators with IgA vasculitis using Mendelian randomization (with SNPs reaching P< 1 x 10-5).

		Inverse variance weighted						MR-Egger					Weighted Median	Weighted Mode	MR-PRESSO
Exposure	No. of SNPs	OR	95% CI	*P*-val	Q	Q *P*-val	I^2	OR	95% CI	*P*-val	intercept	intercept *P*-val	*P*-val	*P*-val	Global Test *P*-val
**Chemokines**															
MIP1b	26	1.143	0.962- 1.359	0.128	22.427	0.611	0.0%	1.049	0.811- 1.357	0.718	0.031	0.387	0.400	0.295	0.655
Eotaxin	25	0.871	0.662 - 1.147	0.325	14.134	0.944	0.0%	0.817	0.446 - 1.496	0.518	0.009	0.816	0.314	0.377	0.949
MCP1	26	0.979	0.724 - 1.322	0.888	29.496	0.244	15.2%	1.043	0.517 - 2.103	0.908	-0.011	0.845	0.949	0.911	0.278
MIG	17	0.994	0.768 - 1.286	0.962	11.363	0.787	0.0%	0.785	0.471 - 1.310	0.369	0.055	0.313	0.959	0.743	0.804
IP-10	15	1.099	0.816 - 1.481	0.533	15.337	0.356	8.7%	0.999	0.512 - 1.950	0.998	0.018	0.757	0.496	0.537	0.363
CTACK	17	1.253	0.981 - 1.599	0.071	11.980	0.745	0.0%	1.135	0.703 - 1.831	0.612	0.026	0.645	0.145	0.250	0.768
RANTES	15	0.992	0.734 - 1.342	0.960	15.745	0.329	11.1%	0.536	0.255 - 1.127	0.124	0.127	0.102	0.382	0.567	0.298
MIP1a	16	1.192	0.898 - 1.583	0.225	7.266	0.950	0.0%	1.130	0.597 - 2.139	0.713	0.009	0.858	0.644	0.997	0.944
GROa	17	1.055	0.873 - 1.273	0.582	15.981	0.454	0.0%	1.115	0.721 - 1.721	0.632	-0.017	0.784	0.793	0.712	0.562
SDF1a	17	1.220	0.777 - 1.915	0.388	12.763	0.690	0.0%	1.300	0.492 - 3.432	0.604	-0.008	0.887	0.988	0.770	0.647
MCP3	10	1.061	0.867 - 1.298	0.564	8.107	0.523	0.0%	1.234	0.742 - 2.054	0.441	-0.048	0.544	0.216	0.415	0.532
**Growth factors**															
SCGFb	24	0.942	0.769 - 1.152	0.559	12.262	0.966	0.0%	0.988	0.681 - 1.432	0.949	-0.012	0.767	0.322	0.371	0.968
PDGFbb	16	1.077	0.802 - 1.446	0.624	9.652	0.841	0.0%	1.054	0.587 - 1.894	0.862	0.003	0.937	0.875	0.912	0.892
SCF	16	1.054	0.702 - 1.581	0.801	9.795	0.832	0.0%	1.501	0.559 - 4.033	0.434	-0.044	0.454	0.809	0.891	0.858
GCSF	14	0.877	0.582 - 1.321	0.530	12.849	0.460	0.0%	0.615	0.291- 1.301	0.228	0.051	0.290	0.461	0.457	0.462
VEGF	19	0.934	0.731 - 1.193	0.582	25.651	0.108	29.8%	0.934	0.619 - 1.408	0.747	0.000	1.000	0.261	0.312	0.189
HGF	12	1.158	0.729 - 1.839	0.534	9.716	0.556	0.0%	2.000	0.701 - 5.705	0.224	-0.074	0.281	0.179	0.317	0.569
MCSF	13	1.192	0.945 - 1.503	0.139	11.444	0.491	0.0%	1.708	1.116 - 2.615	0.031	-0.131	0.074	0.128	0.244	0.484
bNGF	13	0.889	0.649 - 1.216	0.460	12.104	0.437	0.9%	0.696	0.169 - 2.853	0.625	0.035	0.734	0.495	0.451	0.431
FGFBasic	14	1.070	0.680 - 1.682	0.770	11.252	0.590	0.0%	1.265	0.349 - 4.588	0.727	-0.019	0.790	0.880	0.697	0.594

**Interleukins**															
IL-18	18	0.815	0.648 - 1.0242	0.079	19.133	0.321	11.1%	0.884	0.534 - 1.463	0.637	-0.019	0.727	0.450	0.562	0.353
IL-12p70	17	0.856	0.659 - 1.112	0.244	9.413	0.895	0.0%	0.897	0.569 - 1.413	0.647	-0.010	0.807	0.273	0.348	0.922
IL-10	15	0.860	0.629 - 1.173	0.341	9.585	0.792	0.0%	0.773	0.395 - 1.513	0.466	0.014	0.733	0.332	0.288	0.826
IL-17	16	1.033	0.709 - 1.505	0.865	9.644	0.842	0.0%	1.036	0.471 - 2.279	0.930	-0.001	0.993	0.950	0.904	0.859
IL-7	12	0.966	0.753 - 1.238	0.782	9.421	0.583	0.0%	0.787	0.466 - 1.328	0.390	0.051	0.404	0.287	0.252	0.562
IL-16	13	0.987	0.767 - 1.271	0.922	12.546	0.403	4.4%	0.956	0.596 - 1.534	0.855	0.009	0.874	0.494	0.527	0.410
IL-2	16	1.089	0.803 - 1.477	0.583	20.009	0.172	25.0%	1.410	0.773 - 2.572	0.282	-0.053	0.345	0.294	0.398	0.171
IL-4	13	0.973	0.618 - 1.532	0.906	7.105	0.851	0.0%	0.638	0.249 - 1.631	0.368	0.059	0.336	0.706	0.727	0.856
IL-13	17	0.881	0.718 - 1.081	0.223	13.924	0.604	0.0%	0.809	0.541 - 1.211	0.320	0.022	0.641	0.267	0.242	0.664
IL1ra	12	0.917	0.658 - 1.278	0.670	9.426	0.583	0.0%	0.885	0.357 - 2.192	0.797	0.006	0.935	0.477	0.670	0.579
IL-2ra	15	0.845	0.664 - 1.074	0.168	8.745	0.847	0.0%	0.754	0.503 - 1.129	0.194	0.037	0.504	0.146	0.117	0.844
IL-6	11	0.772	0.472 - 1.264	0.304	6.240	0.795	0.0%	0.803	0.294 - 2.189	0.678	-0.005	0.933	0.365	0.500	0.817
IL-9	15	0.840	0.618 - 1.139	0.261	13.881	0.459	0.0%	0.765	0.369 - 1.588	0.485	0.021	0.787	0.413	0.563	0.498
IL-1b	7	1.522	0.932 - 2.483	0.093	5.858	0.439	0.0%	1.037	0.296 - 3.637	0.957	0.060	0.541	0.110	0.126	0.446
IL-5	11	0.941	0.689 - 1.286	0.703	8.515	0.579	0.0%	1.297	0.662 - 2.542	0.468	-0.073	0.319	0.526	0.527	0.600
IL-8	14	1.422	1.053 - 1.921	0.022	9.057	0.769	0.0%	1.616	0.871 - 2.998	0.154	-0.025	0.651	0.370	0.715	0.678
**Other**															
TNFb	8	1.077	0.842 - 1.376	0.556	5.559	0.592	0.0%	1.408	0.871 - 2.275	0.212	-0.075	0.250	0.296	0.378	0.609
TNFa	10	0.755	0.524 - 1.088	0.132	9.690	0.376	7.1%	0.530	0.265 - 1.062	0.111	0.071	0.278	0.569	0.752	0.385
MIF	15	0.833	0.618 - 1.123	0.231	15.235	0.362	8.1%	1.338	0.763 - 2.349	0.328	-0.106	0.077	0.535	0.733	0.364
TRAIL	22	1.058	0.856 - 1.307	0.601	21.348	0.438	1.6%	1.074	0.811 - 1.423	0.623	-0.006	0.869	0.543	0.387	0.455
IFNg	11	0.805	0.498 - 1.299	0.374	3.839	0.954	0.0%	0.733	0.283 - 1.903	0.540	0.015	0.830	0.686	0.760	0.961

Abbreviations: CI, Confidence interval; OR, Odds Ratio; P-val, P-value; Q, Cochran Q statistics; SNPs, single nucleotide polymorphisms.

OR and 95% CI represent change in odds ratio of IgA vasculitis per 1 SD increase in circulating inflammatory regulators level.

After correcting for multiple comparison, P-value < 0.05 was considered as significant.

Table S12. Details of IgA vasculitis predicting SNPs with circulating inflammatory regulators (with SNPs reaching P<1 x 10-5).

Table S12 displayed in Table S12.xlsx.

Table S13. Association of IgA vasculitis with circulating inflammatory regulators using Mendelian randomization (with SNPs reaching P<1 x 10-5).

		Inverse variance weighted						MR-Egger					Weighted Median	Weighted Mode	MR-PRESSO
Outcomes	No. of SNPs	Beta	95% CI	*P*-val	Q	Q *P*-val	I^2	Beta	95% CI	*P*-val	intercept	intercept *P*-val	*P*-val	*P*-val	Global Test *P*-val
**Chemokines**															
MIP1b	17	0.000	-0.026 - 0.026	0.981	19.819	0.228	19.3%	-0.005	-0.064-0.053	0.860	0.003	0.834	0.616	0.566	0.229
Eotaxin	17	0.014	-0.011-0.038	0.266	16.952	0.389	5.6%	0.044	-0.008-0.096	0.117	-0.016	0.218	0.203	0.303	0.390
MCP1	17	0.015	-0.009-0.038	0.218	13.402	0.643	0.0%	0.020	-0.031-0.071	0.460	-0.003	0.828	0.485	0.833	0.674
MIG	17	-0.014	-0.052-0.024	0.468	19.155	0.261	16.5%	-0.014	-0.126-0.034	0.280	0.017	0.391	0.795	0.943	0.278
IP-10	17	0.005	-0.044-0.033	0.786	19.624	0.238	18.5%	-0.017	-0.101-0.066	0.692	0.006	0.756	0.927	0.982	0.258
CTACK	16	-0.017	-0.060-0.026	0.442	19.917	0.175	24.7%	0.046	-0.066-0.158	0.434	-0.030	0.254	0.480	0.799	0.204
RANTES	17	0.0158	-0.026-0.058	0.462	21.817	0.149	26.7%	0.060	-0.026-0.147	0.192	-0.024	0.268	0.194	0.326	0.190
MIP1a	17	0.004	-0.036-0.045	0.838	21.021	0.178	23.9%	-0.002	-0.0894-0.085	0.964	0.003	0.874	0.521	0.663	0.184
GROa	16	-0.001	-0.044-0.042	0.969	19.318	0.200	22.4%	0.039	-0.076-0.154	0.519	-0.019	0.477	0.780	0.482	0.206
SDF1a	17	-0.006	-0.030-0.019	0.652	9.051	0.911	0.0%	-0.006	-0.061-0.049	0.840	0.000	0.995	0.893	0.898	0.910
MCP3	16	0.041	-0.027-0.110	0.236	8.150	0.918	0.0%	0.136	-0.039-0.312	0.151	-0.046	0.269	0.281	0.256	0.923
**Growth factors**															
SCGFb	17	-0.009	-0.043-0.026	0.620	14.629	0.552	0.0%	-0.018	-0.091-0.055	0.640	0.005	0.788	0.669	0.838	0.553
PDGFbb	17	0.022	-0.001-0.046	0.061	9.528	0.890	0.0%	0.037	-0.015-0.088	0.183	-0.007	0.551	0.114	0.364	0.902
SCF	17	0.016	-0.007-0.039	0.172	6.998	0.973	0.0%	0.011	-0.039-0.062	0.669	0.003	0.836	0.314	0.867	0.976
GCSF	17	0.006	-0.018-0.030	0.606	15.214	0.509	0.0%	-0.003	-0.055-0.049	0.912	0.005	0.700	0.982	0.857	0.508
VEGF	17	0.007	-0.018-0.032	0.592	11.423	0.783	0.0%	0.018	-0.038-0.073	0.540	-0.006	0.673	0.298	0.327	0.784
HGF	17	0.008	-0.016-0.033	0.496	17.389	0.361	8.0%	-0.044	-0.096-0.007	0.109	0.028	0.038	0.761	0.708	0.369

MCSF	16	-0.030	-0.076-0.015	0.192	9.105	0.872	0.0%	-0.061	-0.181-0.058	0.330	0.015	0.589	0.106	0.213	0.872
bNGF	17	-0.013	-0.048-0.022	0.456	14.440	0.566	0.0%	-0.053	-0.127-0.021	0.178	0.021	0.248	0.546	0.403	0.562
FGFBasic	17	-0.002	-0.025-0.0286	0.907	19.667	0.236	18.6%	0.019	-0.041-0.079	0.552	-0.009	0.542	0.900	0.262	0.214
**Interleukins**															
IL-18	16	0.019	-0.062-0.087	0.743	14.352	0.499	0.0%	0.013	-0.062-0.087	0.743	0.004	0.840	0.706	0.923	0.510
IL-12p70	17	0.006	-0.017-0.029	0.610	11.146	0.800	0.0%	0.007	-0.044-0.058	0.795	0.012	0.500	0.256	0.408	0.636
IL-10	17	0.012	-0.012-0.036	0.342	7.455	0.963	0.0%	0.023	-0.029-0.075	0.409	-0.006	0.647	0.824	0.976	0.966
IL-17	17	0.01313701	-0.011-0.037	0.285	12.587	0.703	0.0%	0.025	-0.028-0.077	0.373	-0.006	0.637	0.178	0.269	0.725
IL-7	17	0.013	-0.022-0.049	0.469	3.705	0.999	0.0%	-0.010	-0.084-0.065	0.803	0.012	0.505	0.689	0.718	0.999
IL-16	17	0.005	-0.031-0.041	0.786	15.130	0.515	0.0%	-0.024	-0.099-0.052	0.544	0.015	0.409	0.772	0.668	0.507
IL-2	17	0.006	-0.033-0.044	0.777	6.796	0.963	0.0%	0.040	-0.061-0.140	0.452	-0.016	0.483	0.963	0.994	0.972
IL-4	17	0.016	-0.008-0.039	0.197	9.593	0.887	0.0%	0.014	-0.037-0.066	0.594	0.001	0.959	0.990	0.758	0.891
IL-13	17	0.025	-0.010-0.0560	0.170	11.146	0.800	0.0%	0.002	-0.072-0.076	0.960	0.012	0.500	0.461	0.504	0.815
IL1ra	17	0.023	-0.012-0.058	0.198	11.829	0.756	0.0%	0.016	-0.058-0.089	0.680	0.004	0.829	0.418	0.872	0.757
IL-2ra	17	-0.017	-0.057-0.022	0.389	20.746	0.188	22.9%	-0.091	-0.166--0.017	0.030	0.040	0.043	0.197	0.177	0.165
IL-6	17	0.019	-0.004-0.043	0.105	6.683	0.979	0.0%	0.033	-0.018-0.085	0.226	-0.007	0.565	0.115	0.328	0.964
IL-9	17	0.028	-0.011-0.068	0.160	20.393	0.203	21.5%	0.024	-0.062-0.109	0.594	0.002	0.907	0.089	0.282	0.215
IL-1b	18	-0.002	-0.030-0.026	0.909	9.567	0.921	0.0%	0.000	-0.063-0.062	0.989	-0.001	0.967	0.971	0.938	0.919
IL-5	16	0.017	-0.022-0.056	0.397	14.242	0.507	0.0%	0.01	-0.098-0.108	0.923	0.006	0.815	0.978	0.759	0.498
IL-8	17	0.018	-0.024-0.061	0.391	22.791	0.119	29.8%	0.02	-0.068-0.114	0.634	-0.002	0.922	0.764	0.924	0.147
**Other**															
TNFb	10	-0.093	-0.178--0.007	0.033	12.332	0.195	27.0%	0.27	-0.169-0.712	0.262	-0.136	0.139	0.233	0.645	0.216

TNFa	17	0.032	-0.008-0.073	0.117	20.397	0.203	21.6%	0.04	-0.045-0.128	0.358	-0.005	0.808	0.066	0.246	0.203
MIF	15	0.008	-0.031-0.046	0.698	5.418	0.979	0.0%	-0.032	-0.133-0.069	0.545	0.019	0.420	0.727	0.714	0.975
TRAIL	18	0.000	-0.024-0.024	0.990	18.665	0.348	8.9%	0.02	-0.037-0.070	0.556	-0.009	0.512	0.942	0.752	0.351
IFNg	17	0.015	-0.009-0.040	0.213	12.358	0.719	0.0%	0.04	-0.013-0.092	0.163	-0.013	0.329	0.616	0.824	0.698

Abbreviations: CI, Confidence interval; P-val, p-value; Q, Cochran Q statistics; SNPs, single nucleotide polymorphisms.

Beta and 95% CI represent change in SD of circulating inflammatory regulators per log odds increase in IgA vasculitis.

After correcting for multiple comparison, P-value< 0.05 was considered as significant.
